# Diverse haplotypes at a complex *Solanum americanum* locus confer resistance to *Phytophthora infestans* and *P. capsici*

**DOI:** 10.1093/plcell/koag264

**Published:** 2026-09-05

**Authors:** Robert Heal, Andrea Carolina Olave-Achury, Maria Sindalovskaya, Ruth Norkor Annang, Shuting Sun, He Zhao, Qing Liu, Li Long, Hari Sharan Karki, Jodie Taylor, Aleksandra Wawryk-Khamdavong, Kinga Bachowska, Matthew Smoker, Maheen Alam, Sarah Pottinger, Vatsal Arora, Kee Hoon Sohn, Kamil Witek, Xiao Lin, Jonathan D G Jones

**Affiliations:** The Sainsbury Laboratory, University of East Anglia, Norwich Research Park, Colney Lane, Norwich NR4 7UH, United Kingdom; The Sainsbury Laboratory, University of East Anglia, Norwich Research Park, Colney Lane, Norwich NR4 7UH, United Kingdom; Earlham Institute, Norwich Research Park, Norwich NR4 7UZ, United Kingdom; The Sainsbury Laboratory, University of East Anglia, Norwich Research Park, Colney Lane, Norwich NR4 7UH, United Kingdom; John Innes Centre, Norwich Research Park, Colney Lane, Norwich NR4 7UH, United Kingdom; The Sainsbury Laboratory, University of East Anglia, Norwich Research Park, Colney Lane, Norwich NR4 7UH, United Kingdom; The Sainsbury Laboratory, University of East Anglia, Norwich Research Park, Colney Lane, Norwich NR4 7UH, United Kingdom; The Sainsbury Laboratory, University of East Anglia, Norwich Research Park, Colney Lane, Norwich NR4 7UH, United Kingdom; State Key Laboratory of Microbial Diversity and Innovative Utilization, Department of Agri-Microbiomics and Biotechnology, Institute of Microbiology, Chinese Academy of Sciences, Beijing 100101, China; The Sainsbury Laboratory, University of East Anglia, Norwich Research Park, Colney Lane, Norwich NR4 7UH, United Kingdom; The Sainsbury Laboratory, University of East Anglia, Norwich Research Park, Colney Lane, Norwich NR4 7UH, United Kingdom; The Sainsbury Laboratory, University of East Anglia, Norwich Research Park, Colney Lane, Norwich NR4 7UH, United Kingdom; The Sainsbury Laboratory, University of East Anglia, Norwich Research Park, Colney Lane, Norwich NR4 7UH, United Kingdom; The Sainsbury Laboratory, University of East Anglia, Norwich Research Park, Colney Lane, Norwich NR4 7UH, United Kingdom; The Sainsbury Laboratory, University of East Anglia, Norwich Research Park, Colney Lane, Norwich NR4 7UH, United Kingdom; The Sainsbury Laboratory, University of East Anglia, Norwich Research Park, Colney Lane, Norwich NR4 7UH, United Kingdom; The Sainsbury Laboratory, University of East Anglia, Norwich Research Park, Colney Lane, Norwich NR4 7UH, United Kingdom; The Sainsbury Laboratory, University of East Anglia, Norwich Research Park, Colney Lane, Norwich NR4 7UH, United Kingdom; Department of Agricultural Biotechnology, Seoul National University, Seoul 08826, Republic of Korea; Global Plant Immunity Research Center, Seoul National University, Seoul 08826, Republic of Korea; Research Institute of Agriculture and Life Sciences, Seoul National University, Seoul 08826, Republic of Korea; Plant Genomics and Breeding Institute, Seoul National University, Seoul 08826, Republic of Korea; Plant Health Center, Seoul National University, Seoul 08826, Republic of Korea; The Sainsbury Laboratory, University of East Anglia, Norwich Research Park, Colney Lane, Norwich NR4 7UH, United Kingdom; 2Blades, Evanston, IL 60201, United States; The Sainsbury Laboratory, University of East Anglia, Norwich Research Park, Colney Lane, Norwich NR4 7UH, United Kingdom; State Key Laboratory of Microbial Diversity and Innovative Utilization, Department of Agri-Microbiomics and Biotechnology, Institute of Microbiology, Chinese Academy of Sciences, Beijing 100101, China; University of the Chinese Academy of Sciences, Beijing 100049, China; The Sainsbury Laboratory, University of East Anglia, Norwich Research Park, Colney Lane, Norwich NR4 7UH, United Kingdom

## Abstract

Plants encounter diverse pathogens and have evolved a 2-layered innate immune system to detect pathogen molecules and activate defense mechanisms that restrict infection. Most cloned plant *Resistance* (*R*) genes encode NLR immune receptors. *NLR* genes are often found in clusters of paralogs with sequence and copy number variation; whether these *NLR* clusters evolve in response to single or multiple pathogens has been unclear. We report here the isolation of a *Phytophthora capsici* resistance gene, *Rpc2*, along with a novel *P. infestans* resistance gene, *Rpi-amr5*, from 2 *Solanum americanum* accessions. These orthologous genes reside in the *Rpi-amr1* cluster, which has previously been associated with resistance to *P. infestans*. By screening RXLR effector libraries of *P. infestans* and *P. capsici*, we identified multiple effectors recognized by both NLRs. Our findings highlight the complexity of *NLR* clusters and evolution driven by interactions with multiple pathogens. This work will underpin efforts to elevate resistance against *Phytophthora* pathogens and enhances our understanding of NLR evolution.

## Introduction

Oomycete species in the genus *Phytophthora* cause disease in many economically important plant species. Of these, the most notorious pathogen is *Phytophthora infestans*, the causal agent of potato late blight, which triggered the Irish famine in the 1840s and continues to cause significant damage to potato production (Fry 2008; Savary et al. 2019). *P. capsici* is also a highly destructive oomycete pathogen with a broad host range including many important crops such as tomato, pepper, pumpkin, and melon (Kamoun et al. 2015). To date, nearly 50 *Resistance to Phytophthora infestans* (*Rpi*) genes have been cloned (Paluchowska et al. 2022), but little is known about *P. capsici* resistance.

Plant resistance (*R*) genes often encode intracellular nucleotide-binding leucine-rich repeat (NLR) immune receptors, which recognize pathogen effectors and activate effector-triggered immunity (ETI). Most NLRs act as sensors to initiate an immune response after effector detection. While some of these are “functional singletons,” many NLRs require additional “helper” NLRs to activate immunity (Wu et al. 2017; Witek et al. 2021; Lin et al. 2022; Ahn et al. 2023; Contreras et al. 2023; Lin et al. 2023).

Most plant species resist most plant pathogens. This non-host resistance is considered broader and more durable than host resistance (Zimnoch-Guzowska et al. 2003; Schulze-Lefert and Panstruga 2011; Strugala et al. 2015; Lee et al. 2016; Vega-Arreguin et al. 2017; Panstruga and Moscou 2020). *Solanum americanum* is a wild diploid species, which is considered a non-host to *P. infestans* (Colon et al. 1992) and is a useful model for studying plant-microbial interactions and plant immunity. We obtained 54 accessions from gene banks and generated reference-quality genomes for 4 accessions (Lin et al. 2023). By combining map-based cloning, SMRT-RenSeq, BSA-RenSeq, effectoromics, and GWAS, we cloned 5 NLR genes along with their corresponding recognized effectors, including Rpi-amr3/AVRamr3, Rpi-amr1/AVRamr1, Rpi-amr4/AVRamr4, R02860/PITG_02860, and R04373/PITG_04373 (Witek et al. 2016; Lin et al. 2020; Witek et al. 2021; Lin et al. 2022; Lin et al. 2023). Remarkably, no *P. infestans* strains have been found to overcome either Rpi-amr1 or Rpi-amr3 (Witek et al. 2021; Lin et al. 2022). Understanding the durability of these non-host resistance genes will aid in effectively deploying resistance to ensure its longevity in crops. One approach to prevent the breakdown of resistance is the generation of combinations (or stacks) of *R* genes through plant breeding or molecular biology-based approaches. *R* gene stacking has been successfully used to develop late blight resistance in the African potato variety Victoria through the introduction of 3 *R* genes: *Rpi-blb1*, *Rpi-blb2*, and *Rpi-vnt1* (Ghislain et al. 2019). Similarly, 5 stem rust resistance genes (*Sr22*, *Sr35*, *Sr45*, *Sr50*, and *Sr55*) were transferred into the bread wheat cultivar Fielder, resulting in durable resistance without any detectable growth penalty (Luo et al. 2021).

Most characterized oomycete effector genes encode proteins with a signal peptide followed by both RXLR (Arg-X-Leu-Arg) and EER (Glu-Glu-Arg) motifs (Rehmany et al. 2005; Whisson et al. 2007). The *P. infestans* T30-4 reference genome encodes 563 predicted RXLR effectors (Haas et al. 2009; Vleeshouwers et al. 2011). Some effectors lack RXLR or EER motifs but share with many RxLR effectors the presence of WY or LWY domains in the secreted protein (Bailey et al. 2011; Wood et al. 2020; Kim et al. 2024). This has enabled high-throughput effectoromics screening, and led to the identification of multiple *Rpi* and *Avr* genes (Haas et al. 2009; Vleeshouwers et al. 2011; Lin et al. 2023). However, no comprehensive *P. capsici* RXLR effector library has been reported.

Here, we identify and characterize novel *R* genes residing within the complex *S. americanum Rpi-amr1* locus. *Rpi-amr5* was identified from a *P. infestans*-resistant accession lacking functional alleles of *Rpi-amr1* or *Rpi-amr3*. Interestingly, from a *P. capsici* resistant accession, we identified novel alleles of *Rpi-amr5* (named *Resistance to Phytophthora capsici 2*—*Rpc2*), and *Rpi-amr1* (named *Rpi-amr1e-2308*), which confer resistance to both *P. capsici* and *P. infestans*. To investigate the molecular basis of *S. americanum* non-host resistance to *P. infestans*, and its relationship to host resistance to *P. capsici*, we identified effectors recognized by these NLRs. Rpi-amr5 and Rpc2 recognize 2 *P. infestans* effectors. Surprisingly, Rpi-amr5 and Rpc2 also share recognition of 3 *P. capsici* effectors, despite *Rpi-amr5* not conferring resistance to the pathogen. Rpc2 uniquely recognizes the *P. capsici* effector PcE99, which is not recognized by Rpi-amr5. Recognition of PcE99 by Rpc2 likely explains why *Rpc2*, but not *Rpi-amr5*, confers resistance to *P. capsici*.

## Results

### *Rpi-amr5* from *S. americanum* accession SP2275 maps to the *Rpi-amr1* cluster

All tested *S. americanum* accessions resist *P. infestans* in the field, but 5 accessions show susceptibility in detached leaf assays (Lin et al. 2023). *Rpi-amr1* and *Rpi-amr3* were cloned from *S. americanum* accessions SP2273 and SP1102, respectively, and their cognate effectors, AVRamr1 and AVRamr3, were later defined. To identify novel *P. infestans* resistance, resistant *S. americanum* accessions were screened for a lack of response to these 2 effectors. The *S. americanum* accession SP2275 does not recognize AVRamr1, or AVRamr3, but is resistant to *P. infestans*.

**Figure 1:**
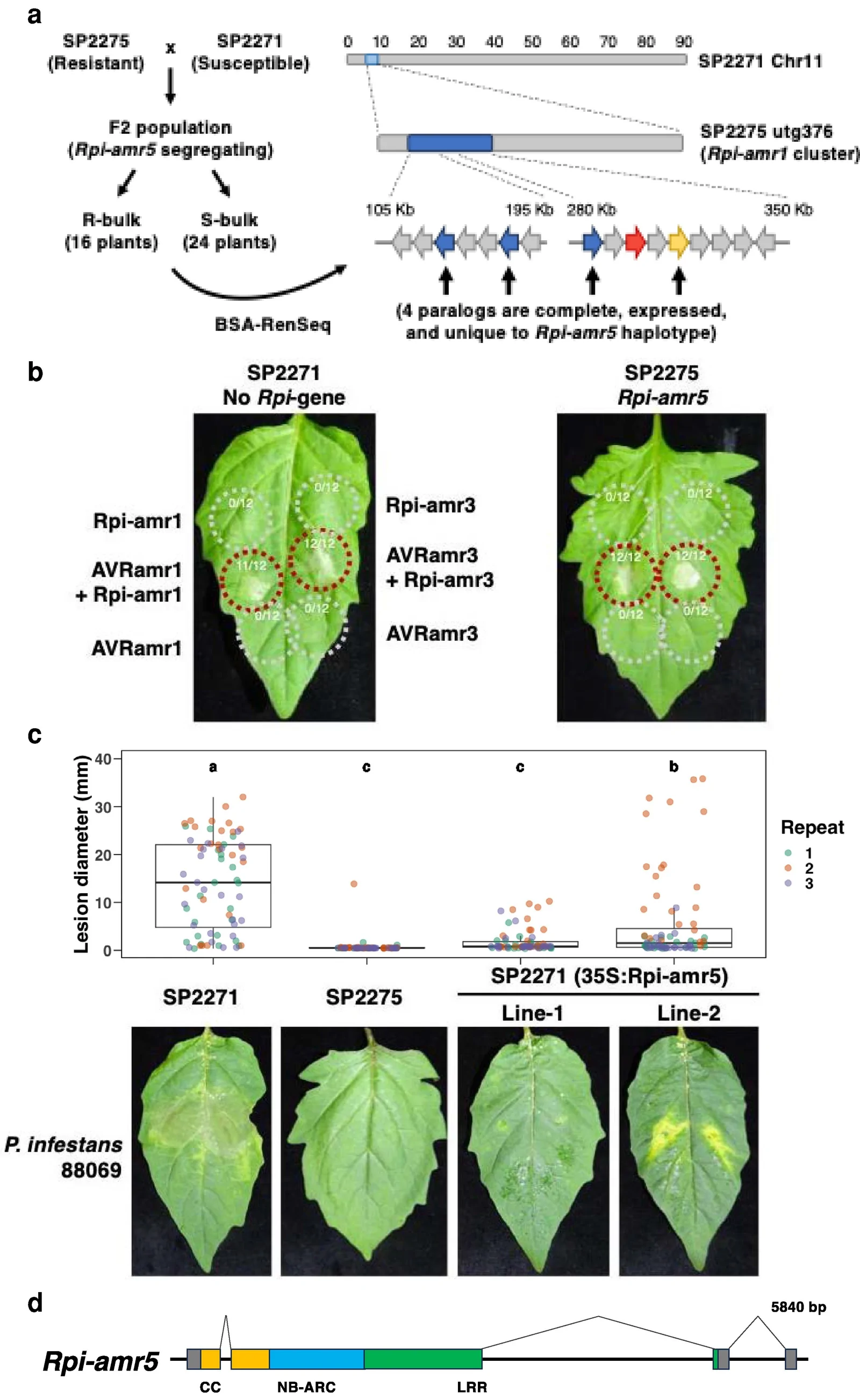
*Rpi-amr5* maps to the *Rpi-amr1* cluster and confers resistance to *P. infestans*. (a) SP2275 was crossed to SP2271, and an F2 population was phenotyped for foliar resistance to *P. infestans*. The 24 most susceptible and 16 most resistant plants were used for bulked segregant analysis using Resistance-gene enrichment sequencing (RenSeq). *Rpi-amr5* was positioned at the *Rpi-amr1* cluster on Chromosome 11, which contains 16 paralogs in SP2275. NLR-encoding genes are represented with arrows, *Rpi-amr5* candidates are indicated in blue. The *Rpi-amr1* ortholog (*Rpi-amr1e*, indicated in red) is non-functional in SP2275 due to a premature stop codon. The paralog corresponding to *Rpi-amr5* is indicated in yellow. (b) *S. americanum* accession SP2275 does not respond to AVRamr1 or AVRamr3. The effectors were transiently expressed in leaves of SP2275 and the susceptible reference accession SP2271. As a positive control for effector expression, the corresponding NLR was transiently co-expressed. *Agrobacterium* strains were infiltrated at an OD_600_ of 0.5; photographs were taken at 3 dpi. (c) Stable transformation with the functional *Rpi-amr5* elevates resistance to *P. infestans* in the susceptible accession SP2271. Leaves were inoculated with 10 μl drops of *P. infestans* zoospores (strain 88069), at a concentration of 20,000 spores mL^−1^. Leaves were imaged at 5 d post-inoculation, and lesion diameters were measured. Plants derived from 2 independent primary transgenic events are shown. Three biological replicates were performed; all data points (72 per treatment) are represented as box-and-whisker plots. Statistical differences were determined using one-way ANOVA and Tukey's HSD test (*P* < 0.05). (d) *Rpi-amr5* is a 4-exon gene which encodes an 897 amino acid CC-NLR with 74.1% identity to Rpi-amr1. Intron and exon structure was determined using cDNA RenSeq data generated from SP2275 leaf tissue, no evidence of alternative splicing was found. The region encoding the CC domain is indicated in yellow, the NB-ARC in blue. and the LRR in green. The 5′ and 3′ UTRs are indicated in grey. The length of *Rpi-amr5* including UTRs, as predicted using cDNA RenSeq data, is shown.

SP2275 was crossed with a susceptible accession (SP2271) yielding resistant F1 plants. In an F2 population, phenotyping revealed a 3:1 (resistant: susceptible) segregation ratio (147 resistant and 53 susceptible; χ^2^(1) = 0.240, *P* = 0.6242), indicating that a single locus is responsible for resistance. To identify NLR-encoding genes linked to resistance in SP2275, 24 susceptible and 16 resistant segregants were used in an approach combining bulked segregant analysis (BSA) and resistance-gene enrichment sequencing (RenSeq) (Fig. 1a). Resistance mapped to the *Rpi-amr1* locus on the short arm of Chromosome 11 (Witek et al. 2021). However, SP2275 does not recognize AVRamr1 (Fig. 1b), therefore this resistance was named *Rpi-amr5*.

To identify *Rpi-amr5*, we annotated the NLR-encoding genes within the *Rpi-amr1* cluster of SP2275. Using NLR-parser (Steuernagel et al. 2015), 16 paralogs within the *Rpi-amr1* cluster of SP2275 were identified. These paralogs form 2 sub-clusters: one was previously identified by Witek et al. (2021) and the other is more distal (Fig. 1a) (Figure S1). Gene models and expression levels of candidates were defined using cDNA RenSeq reads that were mapped to the SP2275 genome. Of the 16 genes in the cluster, 4 are constitutively expressed in SP2275 leaves and encode complete NLRs that have all canonical domains; these were subsequently prioritized as *Rpi-amr5* candidates (Table S1). These genes were named based on homology to NLRs in accession SP2273, where the *Rpi-amr1* cluster was first characterized (Witek et al. 2021) (Figure S1). Clear orthologs were identified for 2 candidates, *Rpi-amr1c-2275* and *Rpi-amr1h-2275*. However, 2 candidates lacked corresponding NLRs in SP2273 and were named based on their position within the SP2275 cluster: NLR_3-2275 and NLR_6-2275. The SP2273 *Rpi-amr1* gene that confers resistance to *P. infestans* is paralog *Rpi-amr1*e; however, its ortholog in SP2275 is non-functional due to a frameshift leading to a premature stop codon (Figure S2). This is consistent with SP2275's absence of AVRamr1 response (Fig. 1b) and indicates that *Rpi-amr5* is a novel *Rpi* gene against *P. infestans*.

### VIGS reveals a high-priority candidate for the paralog that confers *Rpi-amr5* function

To test the function of the 4 *Rpi-amr5* candidates, their open reading frames were amplified from SP2275 gDNA and cloned into expression vectors under the CaMV *35S* promoter. However, *Agrobacterium*-mediated transient expression in *Nicotiana benthamiana* resulted in constitutive, pathogen-independent cell death for 2 candidates, with weaker cell death observed for a third (Figure S3a). As constitutive activity would interfere with gain-of-resistance assays in *N. benthamiana*, we instead used TRV-based virus-induced gene silencing (VIGS) of each paralog combined with assays for loss of resistance in *S. americanum*.

To silence the entire cluster, a fragment from the NB-ARC encoding region of the *Rpi-amr1h*-2275 paralog was cloned into a TRV2 vector, as this sequence is highly conserved among all members of this cluster. Individual paralogs were silenced by targeting sequence-diverged leucine-rich repeat (LRR)-encoding regions (Figure S3b). Silencing of the *Rpi-amr1* family resulted in susceptibility, and silencing of *Rpi-amr1c-2275* alone resulted in a similar phenotype (Figure S3c). While we have not experimentally verified the absence of off-target silencing, the other paralogs share little sequence identity with the VIGS fragment, suggesting that *Rpi-amr1c-2275* is likely the functional gene conferring resistance (Figure S3d, S3e). Furthermore, CRISPR-mediated knock-out of this gene results in loss of *P. infestans* resistance (Heal 2024; Heal et al. 2025). Although off-target mutation in other paralogues was not excluded and VIGS specificity was not experimentally confirmed, together these data identified this paralog as the strongest candidate for *Rpi-amr5*, prompting its further functional characterization.

To test if *Rpi-amr5-2275* provides *Rpi-amr5* function, this gene was stably transformed into the susceptible *S. americanum* accession SP2271, a background in which no constitutive activity was observed following transient expression of *Rpi-amr1c-2275* (Figure S4). Two independent transgenic lines showed elevated late blight resistance (Fig. 1c). *Rpi-amr5* is a 4-exon *Rpi-amr1* paralog that encodes a CC-NLR immune receptor (Fig. 1d). Notably, *Rpi-amr5* is encoded by a distinct paralog within the *Rpi-amr1* cluster, expanding the known functional diversity within this locus.

### Screening *S. americanum* diversity for *P. capsici* resistance reveals *Rpc2* in SP2308

While *S. americanum* has been shown to carry resistance genes for *P. infestans*, *Pseudomonas syringae*, and *Ralstonia solanacearum* (Witek et al. 2016; Moon et al. 2021; Kim et al. 2025), resistance to *P. capsici* has not been previously explored. Screening of 52 *S. americanum* accessions revealed strong resistance in accession SP2308. This accession belongs to *S. americanum* group 4, which shares characteristics with *Solanum nigrescens*, another diploid Solanum species. SP2308 was crossed with the susceptible accession SP2271. An F2 population of 185 plants segregated in a 3:1 (resistant: susceptible) ratio (140 resistant and 45 susceptible; χ^2^ (1) = 0.045, *P* = 0.8319), indicating that resistance is controlled by a single locus. This locus was named *Resistance to Phytophthora capsici 2* (*Rpc2*) (Figure S5a).

### *Rpc2* maps to the *Rpi-amr1*/*Rpi-amr5* cluster

To map *Rpc2*, short-read RenSeq was performed on bulked DNA from 34 susceptible and 30 resistant plants from the phenotyped F2 population. Bulked segregant analysis positioned *Rpc2* within the *Rpi-amr1*/*Rpi-amr5* cluster. Fine mapping using 78 susceptible segregants further narrowed the interval to 508 Kb (Figure S5a). As no reference genome is available for SP2308, we used PacBio RenSeq data to identify *Rpc2* candidates with the closest homology to genes within the corresponding region of the SP2273 *Rpi-amr1* cluster. We identified 5 complete NLR-encoding genes and retained these as *Rpc2* candidates. These genes—*Rpi-amr1a-2308*, *Rpi-amr1c-2308, Rpi-amr1e-2308, Rpi-amr1h-2308,* and *Rpi-amr1l-2308*—were named following the nomenclature described previously (Figure S5b). The candidates include alleles of *Rpi-amr1* and *Rpi-amr5*. Subsequent testing was conducted to identify the functional *Rpc2* gene.

### *Rpc2* is an allele of *Rpi-amr5; Rpi-amr1e-2308* also confers resistance to *P. capsici*

**Figure 2:**
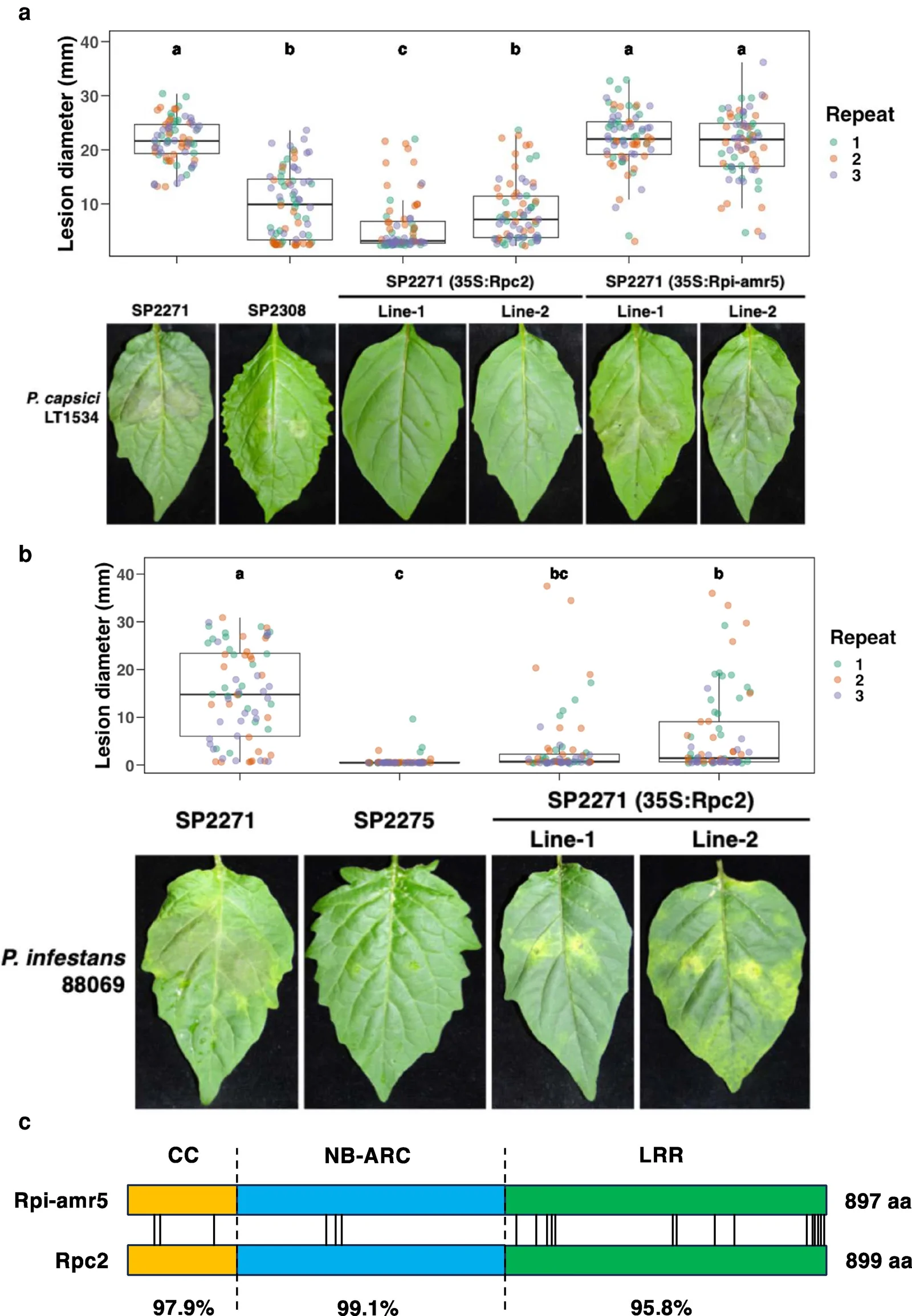
*Rpc2* is the *Rpi-amr5* allele in SP2308 and confers resistance to both *P. infestans* and *P. capsici* in *S. americanum.* (a) Overexpression of *Rpc2*, but not *Rpi-amr5*, elevates resistance to *P. capsici* in the susceptible *S. americanum* SP2271 background. Leaves were inoculated with *P. capsici* mycelial plugs (strain LT1534). At 3 d post-inoculation, lesion diameters were measured and leaves were imaged. Plants derived from 2 independent transgenic events are shown. Three biological replicates were performed, all data points (72 per treatment) are represented as box-and-whisker plots. Statistical differences were determined using one-way ANOVA and Tukey's HSD test (*P* < 0.05). (b) Overexpression of *Rpc2* elevates resistance to *P. infestans* in the susceptible *S. americanum* SP2271 background. Leaves were inoculated with 10 μl drops of *P. infestans* strain 88069 (20,000 spores ml^−1^). At 5 d post-inoculation, lesion diameters were measured and leaves were imaged. Two independent transgenic events are shown. Three biological replicates were performed; all data points (72 per treatment) are represented as box-and-whisker plots. Statistical differences were determined using one-way ANOVA and Tukey's HSD test (*P* < 0.05). (c) *Rpc2* and *Rpi-amr5* are alleles and have highly similar predicted amino acid sequences (97.4% identical). The positions of polymorphisms between the 2 predicted sequences are indicated.

Five *Rpc2* candidates were initially tested in *N. benthamiana* transient assays. Two of these NLRs, including the *Rpi-amr5* ortholog, conferred constitutive HR in *N. benthamiana* (Figure S6a). To further investigate *Rpc2*-mediated resistance, VIGS was used to test functionality of the candidates in the native accession. Silencing the SP2308 *Rpi-amr5* allele (*Rpi-amr1c-2308*) resulted in susceptibility to *P. capsici* (Figure S6b). Furthermore, CRISPR-mediated mutation of this gene results in loss of *P. capsici* resistance (Heal 2024; Heal et al. 2025). Although off-target silencing or mutation cannot be excluded, these data suggest that this paralog is responsible for *P. capsici* resistance in SP2308. Consistent with this, transformation of *Rpi-amr1c-2308* into the susceptible *S. americanum* accession SP2271 conferred resistance to both *P. capsici* and *P. infestans* (Fig. 2a and b). These findings confirm that the primary source of resistance to *P. capsici* in SP2308 is *Rpi-amr1c-2308*, which we designate as *Rpc2*.

**Figure 3:**
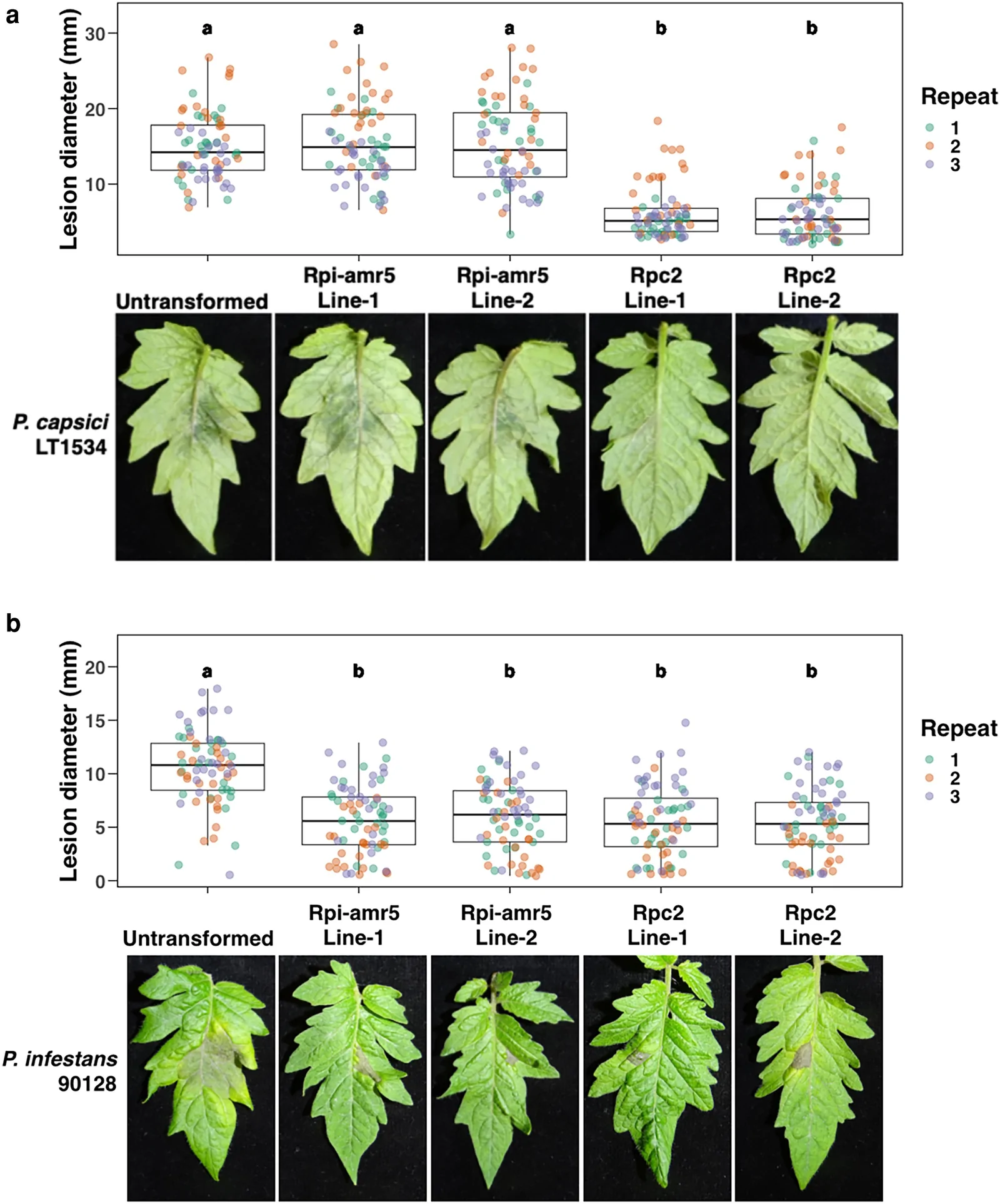
*Rpc2* and *Rpi-amr5* confer *P. infestans* resistance in transgenic *S. lycopersicum*; only *Rpc2* confers resistance to *P. capsici*. (a) *S. lycopersicum* (variety Moneymaker) lines expressing *Rpc2* under *SaNRC4a* regulatory elements are resistant to *P. capsici*, whereas *Rpi-amr5* transgenic lines show no increased resistance compared to the untransformed control. Two independent representative transgenic lines for each construct are shown. Leaves were inoculated with *P. capsici* (strain LT1534) mycelial plugs. At 3 d post-inoculation, lesion diameters were measured and leaves were imaged. Three biological replicates were performed; all data points (72 per treatment) are represented as box-and-whisker plots. Statistical differences were determined using one-way ANOVA and Tukey's HSD test (*P* < 0.05). (b) *S. lycopersicum* transgenic lines expressing either *Rpi-amr5* or *Rpc2* are resistant to *P. infestans*, while the untransformed control is susceptible. Leaves were inoculated with 10 μl drops of *P. infestans* strain 90128 (2,000 spores mL^−1^). At 5 d post-inoculation, lesion diameters were measured and leaves were imaged. Three biological replicates were performed; all data points (72 per treatment) are represented as box-and-whisker plots. Statistical differences were determined using one-way ANOVA and Tukey's HSD test (*P* < 0.05).

*Rpc2* and *Rpi-amr5* share 97.4% amino acid identity (Fig. 2c). While both confer resistance to *P. infestans* in SP2308 and SP2275, respectively, only *Rpc2* confers resistance to *P. capsici* (Fig. 2a). We tested *Rpi-amr5* and *Rpc2* function in *Solanum lycopersicum*, which is susceptible to both *P. infestans* and *P. capsici*. The genes were recloned into a vector containing *SaNRC4a* promoter and terminator to ensure moderate expression levels (Figure S7a). These constructs were transformed into the susceptible tomato variety Moneymaker, and expression was confirmed using RT-PCR (Figure S8). Stable transformants were evaluated through *P. capsici* and *P. infestans* infection assays. Transformants expressing *Rpc2* were resistant to both pathogens, whereas *Rpi-amr5* transformants exhibited resistance only to *P. infestans* (Fig. 3a and b). These results suggest that the inability of *Rpi-amr5* to confer *P. capsici* resistance is likely due to allelic differences between *Rpi-amr5* and *Rpc2*, rather than their expression levels.

**Figure 4:**
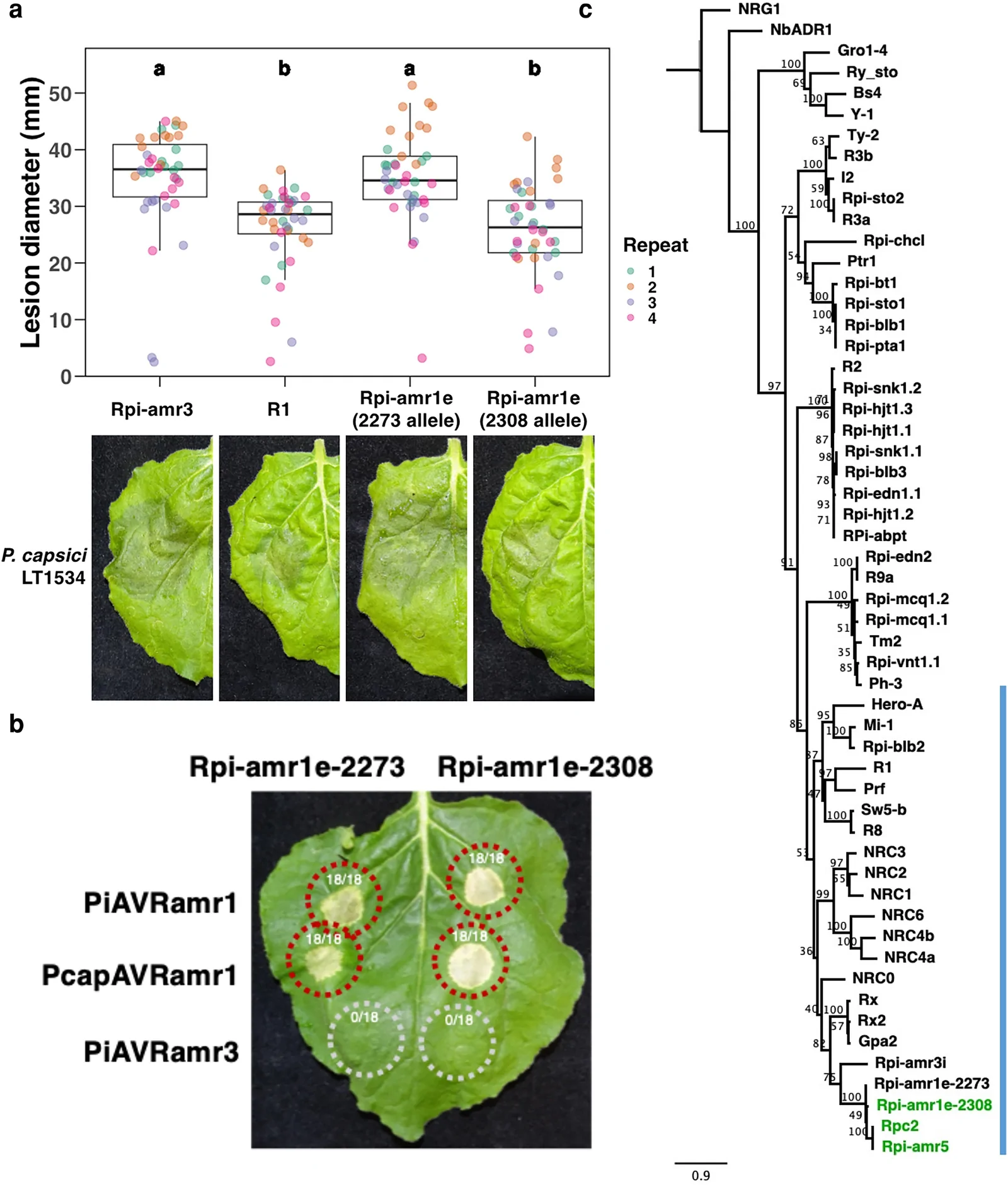
*Rpi-amr1e-2308* also enhances resistance to *P. capsici* in an *N. benthamiana* transient assay. (a) *Rpi-amr1e-2308* confers partial resistance to *P. capsici* in *N. benthamiana* transient assays. *R1* was used as a positive control for *P. capsici* resistance. Agroinfiltration was performed using suspensions normalized to OD_600_ = 0.5. At 2 dpi, leaves were detached and *P. capsici* mycelial plugs were transferred onto them. Lesion size was measured 3 d post-inoculation. Four biological replicates were performed; all data points (40 per treatment) are represented as box-and-whisker plots. Statistical differences determined using one-way ANOVA and Tukey's HSD test (*P* < 0.05). (b) Rpi-amr1-2308 and Rpi-amr1-2273 (with 92.3% amino acid identity) both confer recognition of AVRamr1 orthologs from *P. infestans* (PiAVRamr1) and *P. capsici* (PcapAVRamr1). *N. benthamiana* leaves were infiltrated with *Agrobacterium* suspensions at OD_600_ = 0.2; 2 d later leaves were inoculated. Leaves were imaged at 4 dpi. (c) Phylogenetic tree of Rpi-amr5, Rpc2, Rpi-amr1e-2308, and a panel of previously characterized Solanaceae NLRs. These 3 sensor NLRs are closely related to Rpi-amr1e-2273 and reside within the NRC superclade (indicated by the blue line). The tree was constructed using NB-ARC sequences. Branch support values are shown as percentages, based on 1,000 iterations.

To determine whether other paralogs within the cluster may contribute to resistance in SP2308, we tested three candidates which lacked constitutive activity in *N. benthamiana* (Figure S6a). Leaves expressing these genes were inoculated with *P. capsici* and pathogen growth assessed 72 h post-inoculation. Notably, the SP2308 allele of the late blight resistance gene *Rpi-amr1e (Rpi-amr1e-2308*) significantly reduced *P. capsici* growth compared to *Rpi-amr3* or *Rpi-amr1e-2273*, both of which lack *P. capsici* resistance (Fig. 4a, Figure S9). Interestingly, both *Rpi-amr1e-2308* and *Rpi-amr1e-2273* recognize orthologs of AVRamr1 from *P. infestans* (PiAVRamr1) and *P. capsici* (PcapAVRamr1) (Fig. 4b) (Figure S10, S11). This suggests that *Rpi-amr1e-2308* contributes to *P. capsici* resistance in SP2308.

### Transient expression of *Rpc2* and *Rpi-amr5* with *S. americanum NRC2* enables identification of 2 recognized *P. infestans* effectors

**Figure 5:**
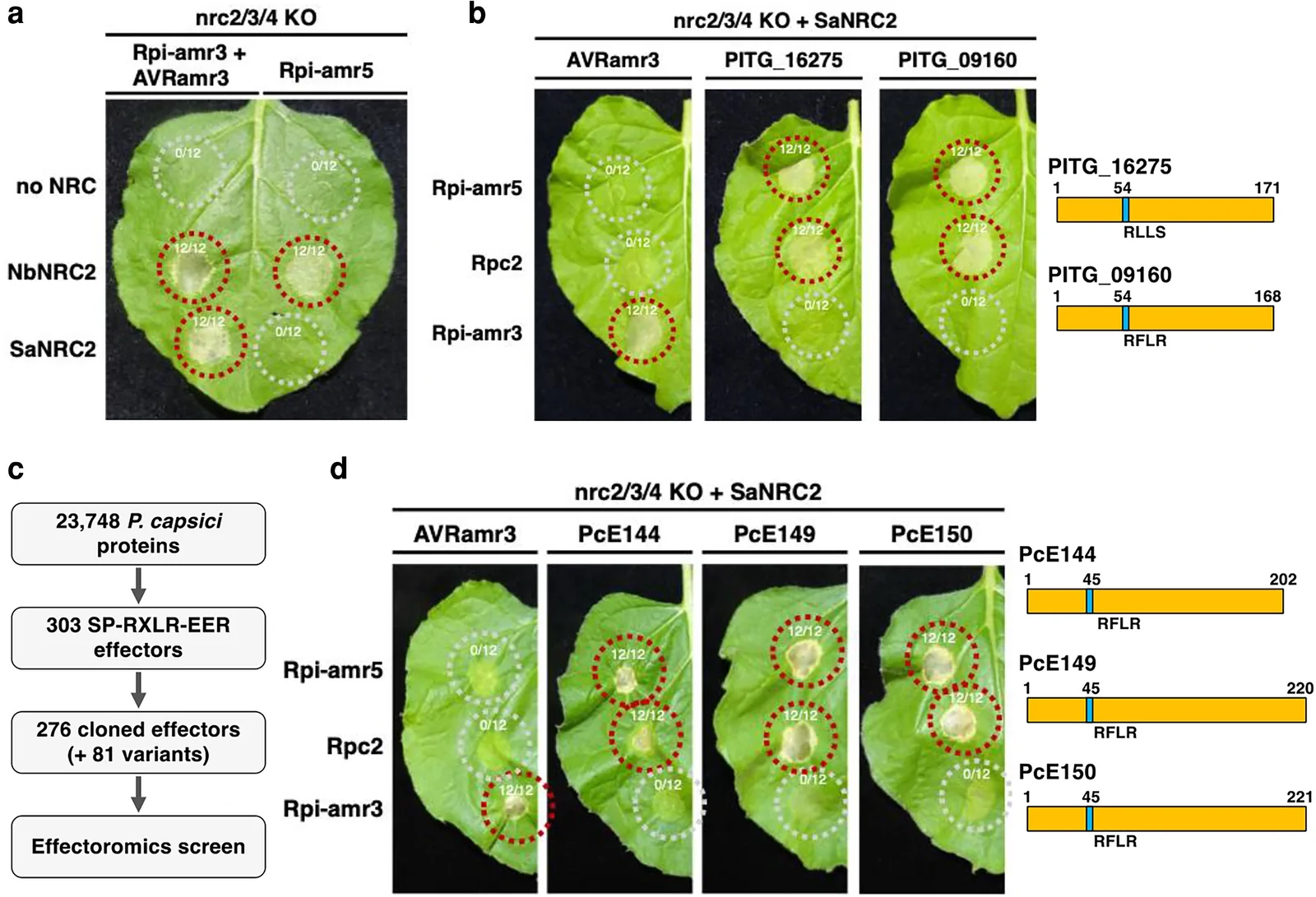
Rpi-amr5 and Rpc2 recognize 2 *P. infestans* effectors and at least 3 *P. capsici* effectors. (a) Absence of NRC helper NLRs abolishes Rpi-amr5/Rpc2 constitutive activity in *N. benthamiana*. Complementation with NbNRC2, but not SaNRC2, restores constitutive activity. *Agrobacterium* strains were infiltrated at OD_600_ = 0.2, and leaves were imaged at 3 dpi. (b) Rpi-amr5 and Rpc2 recognize PITG_09160 and PITG_16275 from *P. infestans*. Co-expression of SaNRC2 with either allele, and either PITG_09160 or PITG_16275 results in effector-dependent HR. Expression of these effectors with Rpi-amr3 and SaNRC2 does not result in cell death. *Agrobacterium* strains were infiltrated at OD_600_ = 0.2, and leaves were imaged at 3 dpi. (c) *P. capsici* effector identification pipeline. From the predicted *P. capsici* proteome, SP-RXLR-EER effectors were identified by filtering for the presence of a signal peptide, RXLR motif, and EER motif. Of the 303 predicted effectors, 276 were cloned. During cloning, 81 additional allelic variants were identified. (d) Rpi-amr5 and Rpc2 both recognize at least 3 *P. capsici* effectors: PcE144, PcE149, and PcE150. Expression of these effectors with SaNRC2 and Rpi-amr5 induces cell death. A similar response was observed with Rpc2. No HR was detected when PcE144, PcE149, or PcE150 were co-expressed with Rpi-amr3. These 3 effectors are typical RXLR-EER-type effectors. All *Agrobacterium* strains were infiltrated at OD_600_ = 0.2. Leaves were imaged at 3 dpi.

Transient expression of *Rpi-amr5* or *Rpc2* under the CaMV *35S* or *SaNRC4a* promoters in *N. benthamiana* results in cell death (Figure S7b). To investigate this effector-independent cell death and enable effector screening, the role of NRC helper NLRs was investigated. Like Rpi-amr1, Rpi-amr5 and Rpc2 are NRC-dependent CC-NLRs (Fig. 4c). NRC proteins are helper NLRs through which cell death is activated after pathogen detection by sensor CC-NLRs (Wu et al. 2017; Contreras et al. 2023). In *nrc2/3/4* mutant *N. benthamiana* plants (Wu et al. 2020), *Rpi-amr5* expression does not trigger cell death. Complementation with *35S:NbNRC2,* but not *35S:SaNRC2* (SaNRC2 = *S. americanum* NRC2), restored strong constitutive activity (Fig. 5a). Additional *NRC* genes cloned from *N. benthamiana* and *S. americanum* were co-expressed with *Rpi-amr5*. Unexpectedly, *Rpi-amr5* also displayed constitutive activity with *NbNRC3* and *SaNRC3* (Figure S12). While the underlying biology is unclear, this finding allowed the co-expression of *35S:SaNRC2* with *Rpc2* or *Rpi-amr5* in *nrc2/3/4* plants for effector screening.

Given the near-identity of Rpi-amr5 and Rpc2, we hypothesized that they recognize the same *P. infestans* effector. All available *S. americanum* accessions have been screened for recognition of 315 *P. infestans* effectors (Lin et al. 2023). Two effectors were recognized by both SP2275 and SP2308, but not SP2271. When transiently expressed in *nrc2/3/4 N. benthamiana* with *35S:SaNRC2* and either *Rpi-amr5* or *Rpc2*, the effector PITG_16275 was recognized by both NLRs (Fig. 5b). Recognition of PITG_16275 by Rpi-amr5 has been independently verified in SP2297, another *S. americanum* accession. Using an F2 population, recognition was mapped to the same locus which contains an *Rpi-amr5* allele encoding an identical NLR (Figure S13). Effectoromics screening (Lin et al. 2023) showed that 24 of the 52 *S. americanum* accessions respond to PITG_16275, suggesting that functional *Rpi-amr5* alleles are widespread in *S. americanum*. Of the 24 accessions that respond to PITG_16275, 19 also respond to AVRamr1. Thus, as observed in SP2273 and SP2275, *Rpi-amr1* and *Rpi-amr5* are likely to be clustered within the same haplotype, forming a natural “stack” of *Rpi* genes in these accessions.

A BLAST sequence homology search for similar *P. infestans* effectors revealed PITG_09160, which shares 39.2% amino acid identity with PITG_16275 after the EER motif. Three additional effectors—PITG_06076, PITG_06059 and PITG_06074—were also identified, but these 3 effectors are not expressed during infection (Lin et al. 2020). Transient expression assays confirmed that *Rpi-amr5* and *Rpc2* recognize both PITG_09160 and PITG_16275, to elicit HR (Fig. 5b). After identifying the *P. infestans* effectors recognized by Rpi-amr5, we tested the dependence of this sensor on SaNRC proteins. In addition to SaNRC2, Rpi-amr5 can be supported by SaNRC1. This contrasts with Rpi-amr1 which is not supported by SaNRC1 (Figure S14) (Lin et al. 2023).

### Homologs of PITG_09160 and PITG_16275 are found in several *Phytophthora* species, but not in *P. capsici*

*Rpi-amr5* and *Rpc2* recognize 2 *P. infestans* effectors, which share similar sequences. We hypothesized that *P. capsici* may contain a similar effector which is recognized by Rpc2. A BLAST search against both the *P. capsici* genome and predicted proteome (Stajich et al. 2021) found no proteins with homology to PITG_16275 or PITG_09160. Therefore, a structural homology search was performed using the bioinformatic tool Foldseek (van Kempen et al. 2024). From the *P. capsici* proteome prediction, 356 effectors were predicted based on the presence of a signal peptide, as well as EER and RXLR motifs (Fig. 5c). AlphaFold3 prediction of these proteins was performed to assemble a library of predicted structures to use as a Foldseek database. However, no proteins were predicted to share high structural similarity with the recognized *P. infestans* proteins (Figure S15).

While no PITG_16275 homologs were identified in *P. capsici*, we identified structurally conserved effectors in *P. palmivora*, *P. parasitica*, *P. megakarya,* and *P. cactorum* (Figure S15). A high-scoring hit from each species was synthesized and transiently expressed with *Rpi-amr5*. Co-expression of the *P. megakarya*, *P. palmivora* and *P. parasitica* orthologs with *Rpi-amr5* caused strong cell death. For the *P. cactorum* ortholog, no clear HR was observed (Figure S16). While resistance to these species has not been tested, this experiment demonstrates that Rpi-amr5 can recognize effectors from at least 5 *Phytophthora* species. Interestingly, many more PITG_16275 homologs were predicted in *P. palmivora* and *P. megakarya* (21 and 28) than in *P. parasitica*, *P. cactorum,* or *P. infestans* (8, 2, and 5), suggesting an expansion of this effector group in these species. Although it remains unclear how many of these homologs are recognized by Rpi-amr5, in combination with the appropriate NRC, this sensor may prove to be a useful resistance gene against these species (Du et al. 2025).

### Three *P. capsici* effectors are recognized by both *Rpi-amr5* and *Rpc2*

No *P. capsici* effectors with homology to PITG_16275 or PITG_09160 were identified using sequence or structural homology searches. Therefore, we cloned a library of 277 FLAG-tagged *P. capsici* RxLR effectors (and 80 additional allelic variants) using a combination of gene synthesis and PCR amplification from *P. capsici* gDNA (Fig. 5c). Effectors were screened by transient expression in SP2308, SP2275 and SP2271. Effectors that elicit HR in SP2308 were then tested for recognition by *Rpc2* and *Rpi-amr5* in *N. benthamiana*. Three *P. capsici* effectors are recognized by Rpc2: PcE144, PcE149, and PcE150. Surprisingly, Rpi-amr5 also showed recognition of these effectors, despite not being able to confer *P. capsici* resistance in *S. americanum* or *S. lycopersicum* (Fig. 5d).

The 3 recognized *P. capsici* effectors have high sequence similarity to each other (65.4% to 92.5% amino acid identity), but low identity to the recognized *P. infestans* effectors (12.2% to 15.0% amino acid identity) (Figure S17). No orthologs of PcE144, PcE149, or PcE150 were identified in *P. infestans*, *P. parasitica*, *P. palmivora*, *P. megakarya,* or *P. cactorum*.

### Rpc2, but not Rpi-amr5, recognizes the *P. capsici* effector PcE99

**Figure 6:**
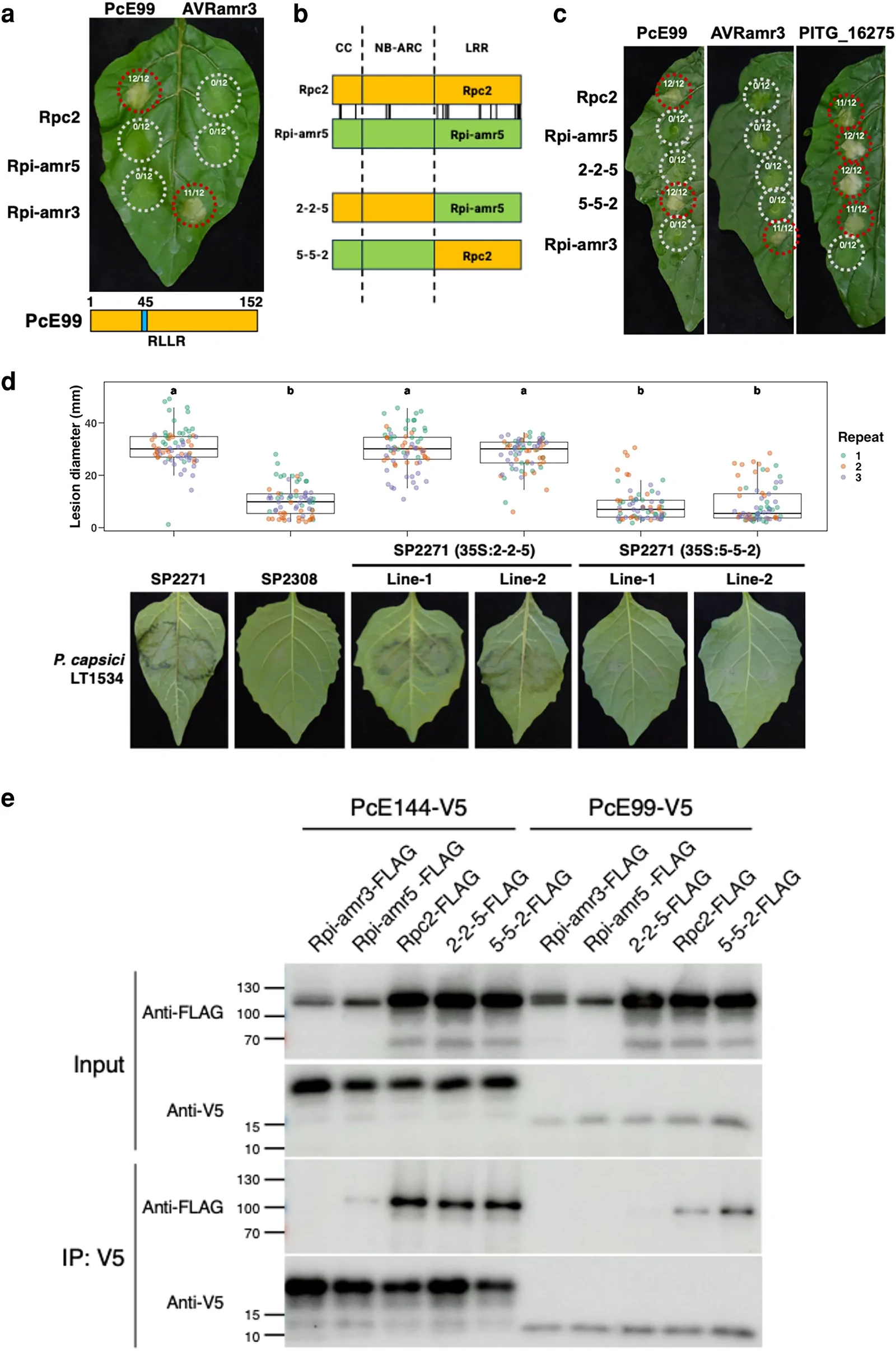
Recognition of the *P. capsici* effector PcE99 is specified by the Rpc2 LRR domain and correlates with resistance to *P. capsici*. (a) Transient expression of Rpc2 with PcE99 in *S. americanum* accession SP2271 results in cell death. No HR was detected when PcE99 was co-expressed with either Rpi-amr5, or Rpi-amr3. (b) To determine whether the differential response to PcE99 between Rpi-amr5 and Rpc2 is due to the LRR domains, reciprocal domain swaps were generated. The positions of polymorphisms the domain exchange are indicated. The resulting chimeras were named 2-2-5, and 5-5-2, as shown. (c) PcE99-induced HR was observed for the 5-5-2 swap, but not the 2-2-5 swap, indicating that recognition of this effector is determined by the LRR domain. Co-expression with AVRamr3 demonstrates that neither chimera is autoactive. Infiltration with PITG_16275 confirms that both chimeras remain functional. *Agrobacterium* strains were infiltrated at OD_600_ = 0.5, and leaves were imaged at 5 dpi. (d) Stable transformation of the 5-5-2 construct into *S. americanum* accession SP2271 confers resistance to *P. capsici*, whereas the 2-2-5 swap does not. Leaves were inoculated with *P. capsici* mycelial plugs (strain LT1534). At 3 d post-inoculation, lesion diameters were measured and leaves were imaged. For each construct, plants derived from 2 independent transgenic events are shown. Three biological replicates were performed; all data points (72 per treatment) are presented as box-and-whisker plots. Statistical differences were determined using one-way ANOVA and Tukey's HSD test (*P* < 0.05). (e) PcE99 co-immunoprecipitates Rpc2 and the 5-5-2 chimera, but not Rpi-amr3, Rpi-amr5, or the 2-2-5 chimera. PcE144 pull-down co-immunoprecipitates Rpi-amr5, Rpc2, and both chimeras, but not Rpi-amr3. Samples were prepared from agroinfiltrated leaves of *nrc2/3/4 N. benthamiana*. *Agrobacterium* strains were infiltrated at OD_600_ = 0.5, and samples were harvested at 2 dpi.

Recognition of PcE144, PcE149, and PcE150 does not correlate with resistance to *P. capsici*, as both Rpi-amr5 and Rpc2 recognize these effectors. We identified another effector, PcE99, that is recognized exclusively in SP2308 and not in either SP2271, or SP2275. Transient co-expression assays in *S. americanum* accession SP2271 demonstrated that PcE99 recognition is mediated by Rpc2 (Fig. 6a).

The LRR domain is commonly implicated in effector recognition specificity in NLR immune receptors, and 19 of 25 polymorphisms between Rpi-amr5 and Rpc2 are located within the LRR-domain (Figure S18). We hypothesized that if differential recognition of PcE99 by Rpc2 and Rpi-amr5 underlies the contrasting resistance phenotypes, then exchanging the LRR domains would alter both recognition and resistance profiles. To test this hypothesis, we generated reciprocal LRR swaps. The Rpi-amr5 CC and NB-ARC domains were combined with the Rpc2 LRR domain (referred to as the 5-5-2 swap), and the Rpc2 CC and NB-ARC domains were combined with the Rpi-amr5 LRR domain (referred to as the 2-2-5 swap) (Fig. 6b). These chimeric NLRs were tested in transient assays to confirm functionality; both the 5-5-2, and 2-2-5 constructs retained the ability to recognize PITG_16275. Notably, HR was observed following co-expression of PcE99 with 5-5-2, but not with 2-2-5 (Fig. 6c), indicating that differential PcE99 recognition is determined by polymorphisms within the LRR domain of Rpc2.

To confirm that PcE99 recognition is responsible for the differential resistance to *P. capsici*, the 5-5-2 and 2-2-5 constructs were stably transformed into the susceptible SP2271. All transgenic lines were resistant to *P. infestans*, confirming that both domain-swaps are functional (Figure S19). We found that 5-5-2 confers resistance to *P. capsici*, whereas 2-2-5 does not, demonstrating that recognition of PcE99 correlates with resistance to *P. capsici* (Fig. 6d).

### Rpi-amr5 and Rpc2 associate with their cognate effectors *in planta*

Rpi-amr5 and Rpc2 share recognition of 5 effectors: 2 from *P. infestans* and 3 from *P. capsici*. These effectors vary significantly in length and amino acid sequence (Fig. 5b and d, Figure S17). A split luciferase assay was conducted to test for *in planta* association between the NLRs and these 5 effectors. In this assay, the NLRs were fused with the C-terminal fragment of luciferase, while effectors were tagged with the N-terminal fragment. Constructs were expressed in the *nrc2/3/4 N. benthamiana* line, to prevent cell death being triggered. Luminescence was observed when any of the 5 recognized effectors were co-expressed with either Rpi-amr5 or Rpc*2*. In contrast, no luminescence was detected upon co-expression with Rpi-amr3, or the control effector AVRamr3, which does not activate Rpi-amr5 or Rpc2 (Figure S20a and b).

To further validate interactions between Rpi-amr5 or Rpc2 and their cognate effectors, we performed co-immunoprecipitation (CoIP) assays using FLAG-tagged NLRs and V5-tagged effectors. V5-immunoprecipitated samples were analyzed by SDS-PAGE. Upon immunoprecipitation of the *P. infestans* effectors PITG_09160-V5 and PITG_16275-V5, both Rpi-amr5 and Rpc2 were detected, whereas Rpi-amr3 was not. These CoIP results support our split luciferase data and suggest that Rpi-amr5 and Rpc2 associate with their cognate *P. infestans* effectors (Figure S20c).

Similarly, CoIP assays were performed using 2 recognized *P. capsici* effectors; PcE144 and PcE99. Both Rpi-amr5 and Rpc2, but not Rpi-amr3, co-immunoprecipitated with PcE144-V5. Consistent with the lack of PcE99 recognition by Rpi-amr5, only Rpc2 was detected following PcE99-V5 immunoprecipitation. Furthermore, the 5-5-2 swap, but not the 2-2-5 swap, co-immunoprecipitated with PcE99 (Fig. 6e).

Together with resistance phenotyping of the domain-swap lines, these results demonstrate that resistance to *P. capsici* is determined by polymorphisms within the Rpc2 LRR domain that confer recognition of and interaction with PcE99. However, it remains unclear why recognition of PcE144, PcE149, and PcE150 by Rpi-amr5 does not translate into resistance to *P. capsici*.

## Discussion

### Several distinct paralogs of the *Rpi-amr1* cluster confer *Phytophthora* resistance

The *Phytophthora* genus contains a diverse range of plant pathogens, many of which have significant economic impact. *P. infestans*, the causal agent of late blight, remains a global threat to potato production, while *P. capsici* causes disease in numerous *Solanum, Capsicum,* and Cucurbitaceae species. While *S. americanum* is a non-host to *P. infestans*, most *S. americanum* accessions are susceptible to *P. capsici*.

**Figure 7:**
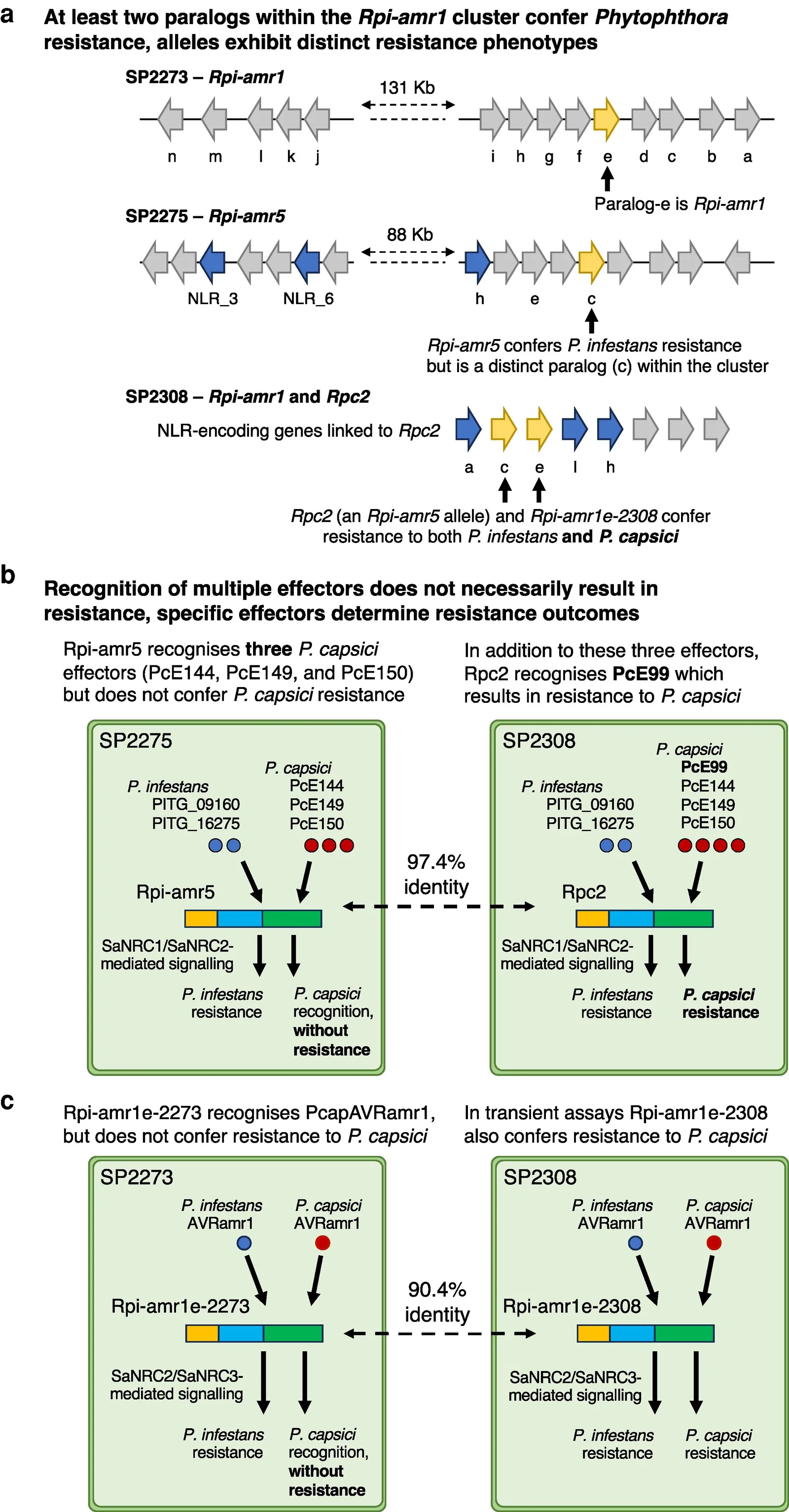
A complex *Solanum americanum* resistance gene locus confers resistance to *Phytophthora infestans* and *P. capsici*. (a) The *Rpi-amr1* clusters in *S. americanum* accessions SP2273 (the source of *Rpi-amr1e-2273*), SP2275 (the source of *Rpi-amr5*), and SP2308 (the source of *Rpc2* and *Rpi-amr1e-2308*) contain distinct genes that confer resistance to *P. infestans* and *P. capsici*. NLR-encoding genes are represented with arrows, genes known to confer resistance to either *P. infestans* or *P. capsici* are indicated in yellow. (b) *Rpi-amr5* and *Rpc2* were cloned from accessions SP2275 and SP2308, respectively. Rpc2 recognizes 2 effectors from *P. infestans* and 3 from *P. capsici*, and confers resistance to both pathogens. *Rpi-amr5* (an allele of *Rpc2*) recognizes the same effectors but does not confer resistance to *P. capsici*. (c) Rpi-amr1e-2273 recognizes AVRamr1 orthologs from both *P. infestans* and *P. capsici*, but does not confer resistance to *P. capsici*. A novel allele, *Rpi-amr1e-2308*, confers both recognition and resistance.

We show here that the *S. americanum* accession SP2275 carries *Rpi-amr5,* which encodes a CC-NLR immune receptor and confers resistance to *P. infestans* (Fig. 7a). Unlike the previously characterized resistance genes *Rpi-amr3*, *Rpi-amr4*, *R02860*, and *R04373*, *Rpi-amr5* is encoded by a previously uncharacterized paralog within the *Rpi-amr1* cluster. In parallel, resistance to *P. capsici* was characterized in *S. americanum* accession SP2308. Two NLR-encoding genes located at the SP2308 *Rpi-amr1* locus confer resistance to both *P. capsici* and *P. infestans*; *Rpc2,* an allele of *Rpi-amr5*, confers most of the *P. capsici* resistance, while weaker resistance is conferred by *Rpi-amr1e-*2308, a novel allele of *Rpi-amr1* (Fig. 7a). Similarly, of 24 *Rpi-amr5*-containing haplotypes, 19 also carry a functional *Rpi-amr1* allele. While we do not know whether these genes are closely linked in every accession, in both SP2273 and SP2275 they are located within the same gene cluster. Together, these findings demonstrate that multiple paralogs within the *Rpi-amr1* cluster confer resistance to *Phytophthora* species and that gene stacks have frequently arisen and been selected.

### Interspecific combinations of helper NLR and sensor NLR can result in effector-independent defense activation

Both Rpi-amr5 and Rpc2 activate constitutive HR in *N. benthamiana*, but not in *nrc2/3/4* knockout lines. This constitutive activity can be restored in *nrc2/3/4* lines by co-expression with *35S:NbNRC2*, but not *35S:SaNRC2*, suggesting that inappropriate combinations of sensor and helper NLRs can be maladaptive. Recent studies have shown that the restricted taxonomic functionality (RTF) of NRC-dependent sensor NLRs results from the absence of a compatible helper NLR (Goh et al. 2024; Du et al. 2025). Our data suggest that in addition, interspecific combinations of sensor NLRs and NRCs can result in aberrant, effector-independent activation of helper NLRs of one species by sensor NLRs from another species. Conceivably, such outcomes could have arisen during introgression of *R* genes from wild relatives of crops, though deleterious combinations would have been selected against by plant breeders. *Rpc2* and *Rpi-amr1e-2308* may have broad utility, either through direct transfer into solanaceous hosts of *P. capsici*, such as tomato and pepper, or by transfer to less related species, such as cucurbits. Cucurbitaceae lack NRC proteins (Wu et al. 2017); however, co-transfer of *Rpc2* and *SaNRC2* into cucurbits could elevate resistance to *P. capsici* (Du et al. 2025).

### Rpi-amr5 and Rpc2 share recognition of multiple *Phytophthora* effectors, but PcE99 recognition is Rpc2-specific

By co-expressing *Rpi-amr5* or *Rpc2* in *nrc2/3/4* lines together with *35S:SaNRC2* and clones from a *P. infestans* effector library, we identified PITG_16275 and PITG_09160 as effectors recognized by Rpi-amr5 and Rpc2. However, no orthologs of PITG_16275 were identified in *P. capsici*. To identify *P. capsici* effectors recognized by Rpc2, we generated and screened a library of RXLR effectors in *S. americanum*. This screen identified 3 *P. capsici* effectors recognized by both Rpi-amr5 and Rpc2. Co-immunoprecipitation, and split luciferase assays suggest that there is close physical proximity of Rpi-amr5/Rpc2 and their recognized effectors *in planta*. While these 2 receptors share recognition of multiple *P. capsici* effectors, they differ in their response to PcE99, with only Rpc2 showing a response in co-expression assays.

### Why does *Rpi-amr5* not confer resistance to *P. capsici*?

Four *P. capsici* effectors are recognized by Rpc2, and unexpectedly, Rpi-amr5 also recognizes 3 of these. Despite this, *Rpi-amr5* does not confer resistance to *P. capsici* (Fig. 7b). Similarly, *Rpi-amr1e-2308* provides resistance to *P. capsici*, whereas the *Rpi-amr1e-2273* allele does not, despite both conferring HR when co-expressed with the *P. capsici* AVRamr1 ortholog (Fig. 7c). This is consistent with previous reports of incomplete correspondence between HR to recognized effectors and disease resistance (Gilroy et al. 2011; Lin et al. 2023; Oh et al. 2024). Notably, *S. americanum* R02860 and R04373 recognize *P. infestans* effectors PITG_02860 and PITG_04373, but neither confer resistance (Lin et al. 2023). Although puzzling, this phenomenon is not unprecedented.

Consistent with the fact that there are 19 polymorphisms in the LRRs compared to only 3 in the CC and 3 in the NB-ARC domains, we demonstrated that resistance to *P. capsici* depends on recognition of PcE99, which is unique to Rpc2. Exchange of the LRR domains between these NLRs showed that resistance to *P. capsici* is conferred by a chimera comprising the Rpi-amr5 CC and NB-ARC domains fused to the Rpc2 LRR domain. This gain of function is supported by the acquisition of PcE99 association in co-immunoprecipitation assays.

The lack of observable resistance despite recognition of PcE144, PcE149, and PcE150 remains unexplained. This may reflect low expression levels of these effectors during infection or a quantitatively weaker association with the NLRs compared to PcE99. This highlights a limitation of co-expression cell-death assays and the effectoromics approach more generally, in which phenotypes are assessed following overexpression *in planta* of each component. Such conditions are unlikely to reflect the abundance, stoichiometry, or timing of expression of these components during natural infection. Consequently, the threshold required to trigger defense activation and cell death may be substantially lower than under the native infection conditions. Transcriptomic or proteomic analyses could test this hypothesis and may provide an additional filtering step when identifying effectors for effectoromics. Alternatively, *P. capsici* may deploy additional effectors that suppress recognition of PcE144, PcE149, and PcE150 *in planta*, while leaving recognition of PcE99 unaffected. It also remains unexplained why even though Rpi-amr1-2308 and Rpi-amr1-2273 both confer HR when co-expressed with PcAVRamr1 (Fig. 4b), only Rpi-amr1-2308 confers *P. capsici* resistance in an *N. benthamiana* transient assay.

### Host and non-host resistance in *S. americanum* directly overlap

Non-host resistance is a multifaceted phenomenon, often involving multiple mechanisms. As highlighted by Panstruga and Moscou (2020), NLR-mediated immunity contributes significantly to both host and non-host resistance. While *S. americanum* is a non-host to *P. infestans* in the field, resistance to *P. capsici* is uncommon, displaying features of host resistance. We show here that resistance to these 2 pathogens involves different haplotypes at the *Rpi-amr1* locus. Two paralogs—*Rpi-amr1* and *Rpi-amr5*—confer resistance to *P. infestans* in both haplotypes. In the *P. capsici* resistant accession SP2308, novel alleles of *Rpi-amr1* (*Rpi-amr1e-2308*) and *Rpi-amr5* (*Rpc2*) also provide resistance to *P. capsici*.

In characterizing NLR-mediated non-host resistance to *P. infestans* in *S. americanum*, we have so far identified 4 *Rpi* genes that confer qualitative resistance: *Rpi-amr1*, *Rpi-amr3, Rpi-amr4,* and *Rpi-amr5.* All *S. americanum* accessions with qualitative resistance to *P. infestans* in detached leaf assays, as described by (Lin et al. 2023), contain at least one of these *Rpi* genes. The broad-spectrum resistance profiles of *Rpi-amr1* and *Rpi-amr3* suggest that they may derive durability from the presence of multiple *Rpi* genes. Further elucidation of the genetic and molecular basis of non-host resistance will aid in deploying resistance strategies that are both sustainable and durable in real-world conditions. Specifically, the transfer of multiple stacked *R* genes into disease-susceptible crop species may provide more durable resistance by replicating the natural gene stacks observed in species such as *S. americanum*. Such an approach could also minimize the risk of resistance breakdown resulting from pathogen diversification or loss of recognized effectors. This highlights the need to identify additional *R* genes against *P. capsici*, as loss of PcE99 may compromise resistance if *Rpc2* were deployed alone.

## Methods

### Constructs for transient and stable transformation

To verify candidate genes, ORFs were amplified and cloned into vectors containing the CaMV 35S promoter and the *Agrobacterium tumefaciens* Ocs terminator, using either GoldenGate or USER cloning as previously described (Witek et al. 2016; Lin et al. 2023). Candidates cloned using GoldenGate were ligated into pICLS86922, while USER clones were generated by ligation into pICSLUS0004OD. Additional constructs were generated to test *Rpi-amr5* and *Rpc2* under *S. americanum NRC4a* (*SaNRC4a*) regulatory elements. A custom vector (pICSLUS0005OD) was generated to accept each ORF following the USER method (Nour-Eldin et al. 2010). This vector includes a 943 bp promoter and a 793 bp terminator, both cloned from *S. americanum* accession SP1102.

*Agrobacterium* suspensions were prepared to OD described in the figure legends. For hypersensitive response assays in *N. benthamiana*, and *S. americanum,* fully expanded leaves were selected from plants aged between 5 and 6 wk. Leaves were spot-infiltrated using 1 mL needleless syringes. When multiple infiltrations were made on a single leaf, care was taken to avoid overlapping infiltration zones. Plants were left for 3 to 5 d to allow cell death to develop. To test candidate *Rpi* and *Rpc* genes in *N. benthamiana*, infiltration of whole leaves was performed. Leaves were left for 24 h before being removed and inoculated as described below.

### Disease assays

Two *P. infestans* strains were used: strain 88069 for infection of *S. americanum* and strain 90128 for infection of *S. lycopersicum*. Both strains were maintained at 17 °C on rye-sucrose agar (RSA) medium. To induce zoospore release, 14-d-old plates were flooded with cold water and incubated at 4 °C for 1 to 2 h. Zoospore concentrations were determined using a hemocytometer before dilution to the appropriate concentration (as indicated in figure legends).

For *P. capsici* infection assays, LT1534 was used. *P. capsici* was maintained on V8 agar plates incubated at 25 °C. To induce zoospore release, 10-d-old plates were flooded with cold water and incubated at 4 °C for 1 h, followed by 1 h at room temperature. The suspension was diluted to 10,000 spores ml^−1^.

Detached leaf assays were used to assess the resistance of *S. americanum* and *S. lycopersicum* to *P. infestans* and *P. capsici*. Fully expanded leaves from 5- to 6-wk-old plants were placed on damp paper within 500 cm^2^ square culture dishes. Leaves were inoculated by either transfer of infected agar plugs (2 to 3 mm^2^ plugs were used in quantitative *P. capsici* infection assays), or by pipetting 10 µL droplets onto each leaf. For *P. infestans* infection assays, trays were incubated at 17 °C for 5 to 10 d until susceptible controls showed infection. For *P. capsici* assays, trays were incubated at 25 °C for 2 to 4 d until susceptible controls showed infection.

### Virus-induced gene silencing of *S. americanum*

VIGS was used to silence candidate *R* genes in *S. americanum*. A BsaI-compatible TRV2 vector was used (Duggan et al. 2021). To silence the *Rpi-amr1*/*Rpi-amr5*/*Rpc2* cluster, a highly conserved 265 bp fragment from the NB-ARC-encoding region of *Rpi-amr1h-2275*. To target individual paralogs, the least conserved regions were identified. These regions were generally shorter (between 51 and 115 bp). For each paralog, 2 separate fragments were used. A conservative approach was used to define off-target silencing: the candidate region was divided into 21-mer sequences, off-target hits were defined as those where fewer than 2 SNPs were present within these 21-mer sequences (ie ≥ 90.5% identity).

VIGS of *S. americanum* was performed by vacuum infiltration of seedlings between 2 and 5 d post-germination. *Agrobacterium* suspensions of TRV1 and TRV2 (with the silencing fragment) were mixed in a 1:1 ratio to a final OD_600_ of 0.3. Before infiltration, acetosyringone (500 µM) and Silwet-L77 (0.1 µL/mL) were added. Seedlings were submerged into the *Agrobacterium* suspension, and a vacuum was applied for 1 min, before being released. This was repeated 2 additional times. Following infiltration, seedlings were transferred to compost and grown in glasshouse conditions. When plants were 5 wk old, leaves were selected and drop-inoculated with either *P. infestans* or *P. capsici* zoospores. To confirm the effectiveness of gene silencing, a fragment from the gene encoding the magnesium-chelatase subunit ChIH was used. Silencing of this gene results in tissue bleaching, acting as a marker for successful VIGS.

### Effectoromics screen

To identify effectors recognized by the genes cloned in this study, RXLR effector libraries were screened on *S. americanum* accessions. A library of 311 *P. infestans* effectors was previously screened on these accessions (Lin et al. 2023). Using the predicted proteome of *P. capsici* (Stajich et al. 2021), a new RXLR effector library was generated based on the presence of a signal peptide, RXLR motif, and EER motif. These effectors were cloned into 35S expression vectors (pICSL86977_OD, pICSL86977OD_Aar1, or pICSLUS0004OD), depending on the presence of BsaI, BbsI, or AarI sites. Effectors cloned via the GoldenGate system were fused with a FLAG tag, those cloned through USER cloning remain untagged. *S. americanum* plants were grown under glasshouse conditions, and plants aged 4- to 5-wk were used for infiltration with *Agrobacterium* suspensions (OD = 0.5). Cell death was assessed at 4 d post-infiltration. For each experiment, 2 leaves from 2 different plants were used. *Rpi-amr3* expressed alone was used as a negative control for cell death. Co-expression of *Rpi-amr3* and *AVRamr3* was used as a positive control. Effectors which elicit HR in lines carrying *Rpi-amr5* and *Rpc2* were rescreened in *N. benthamiana* transient assays to identify true elicitors of these sensor NLRs. These *N. benthamiana* assays were independently repeated at least 3 times, using 4 leaves from 4 plants in each repetition.

### Plant growth and transformation

*S. americanum* transformation was performed as described by Lin et al. (2023). For each transformation, a minimum of 2 independent lines are shown. Transgenic *S. lycopersicum* was generated following the previously published protocol (Fillatti et al. 1987), using the cultivar, Moneymaker.

RT-PCR was used to assess expression of *Rpi-amr5* and *Rpc2* transgenes in stably transformed *S. lycopersicum* lines. RNA was extracted using the QIAGEN RNeasy Plant Mini Kit. Genomic DNA was removed using the TURBO DNA-free TM Kit (ThermoFisher Scientific: AM1907). First-strand cDNA synthesis was performed using the Superscript IV Reverse Transcriptase (ThermoFisher Scientific: 18090010), according to the manufacturer's protocol. An equal amount of cDNA template was used in each reaction to compare transgene expression with an untransformed control. A primer pair targeting *EF1α* was used to assess cDNA template quality. A primer pair targeting *Rpi-amr5* and *Rpc2* was used to confirm transgene expression.

*N. benthamiana* was grown in a controlled environment room under conditions of 22 °C, 45 to 65% relative humidity, and a 16-h light/8-h dark cycle. Both wild type and nrc2/3/4 knockout *N. benthamiana* lines were used (Wu et al. 2020).

### Split luciferase assay

To assess *in planta* association between NLRs and their cognate effectors, a split luciferase assay was performed. NLRs were fused to the C-terminal fragment of luciferase (pICSL50048), while effectors were fused to the N-terminal luciferase fragment (pICSL50047). Coding sequences and tags were cloned into the pICSL86922 vector using GoldenGate assembly. This acceptor contains the CaMV *35S* promoter and *Ocs* terminator. *Agrobacterium* strains carrying these constructs were spot-infiltrated into the *nrc2/3/4 N. benthamiana* line.

Two days post-infiltration, leaves were harvested for imaging. Immediately before imaging, leaves were infiltrated with 100 mM sodium citrate buffer containing 0.4 mM luciferin. Luminescence was visualized using the Nightowl II LB 983 In vivo imaging system and Winlight software (Berthold Technologies, Germany). As a positive control, the *S. americanum* NLR Rpi-amr3 and its cognate effector Avramr3 were used (Witek et al. 2016; Lin et al. 2022).

### Co-immunoprecipitation assays

Co-immunoprecipitation assays were conducted following previously described methods (Ahn et al. 2023). To produce protein *in planta* whole *N. benthamiana* leaves were infiltrated and harvested at 2 d post-infiltration. In this study the effectors were immunoprecipitated using a V5 antibody conjugated to agarose beads (Sigma-Aldrich, A7345) before subsequent detection using both V5 (1:5,000, Sigma-Aldrich, V2260) and Flag (1:10,000, Sigma-Aldrich, A8592) antibodies. The C-terminal tags used in these experiments 3xFlag (pICSL50007) for NLRs, and V5 (pICSL50012) for effectors.

### BSA-RenSeq and map-based cloning

Three F2 mapping populations were used in this study: (i) SP2271 x SP2275, (ii) SP2271 x SP2308, and (iii) SP2271 x SP2297. Population 1 was phenotyped for *P. infestans* resistance, population 2 for *P. capsici* resistance, and population 3 for responsiveness to transient expression of PITG_16275. Genomic DNA was extracted from leaf tissue using the Qiagen DNeasy plant kit (Qiagen, 69104). RenSeq libraries were then prepared using bulked genomic DNA from resistant and susceptible individuals. Enrichment for NLR-encoding genes was performed using the V4 RenSeq bait library (Witek et al. 2021). Libraries were sequenced using paired 250 bp illumina sequencing by Novogene (Beijing, China). Bulked segregant analysis was performed following previously described methods (Jupe et al. 2013; Andolfo et al. 2014; Witek et al. 2016). Resistance-linked *R* gene candidates were filtered based on the presence of motifs identified using NLR-parser, and, when available, by their expression as determined using cDNA RenSeq. To detect expression, cDNA RenSeq reads were mapped using HISAT spliced aligner under default settings (Kim et al. 2015). BAM files were visualized using IGV to assess expression and detect splice variants. RNA used for cDNA RenSeq was isolated from uninfected leaves taken from 5-wk-old plants. Consequently, candidates selected for testing were restricted to those that are constitutively expressed.

### Annotation of NLRs and gene models

The NLR-encoding genes either within mapping intervals, or on resistance-linked RenSeq contigs were predicted using NLR-parser (Steuernagel et al. 2015). To generate accurate gene models, cDNA RenSeq reads were mapped to reference sequences as described above. Resulting BAM files were manually inspected to annotate NLR-encoding genes. Published sequences and gene models for *S. americanum* genomes were used to support the manual annotation.

Phylogenetic analyses were performed to establish the relationship between candidate NLRs and previously published receptors. NB-ARC sequences were extracted and aligned using MAFFT. Phylogenetic trees were generated using RAxML, with branch support assessed using 1,000 bootstrap replicates. Default settings were used.

## Supplementary Material

koag264_Supplementary_Data

## Data Availability

The BSA-RenSeq data used to clone *Rpi-amr5* and *Rpc2* are deposited in ENA under BioProject number PRJEB126537. The *Rpi-amr5* and *Rpc2* sequences were deposited at NCBI GenBank under accession number PZ859215 and PZ859216.
