# Supplementary material for: Diverse haplotypes at a complex Solanum americanum locus confer resistance to Phytophthora infestans and P. capsici
Source: Plant Cell. 2026 Sep 5;38(9):koag264. doi: 10.1093/plcell/koag264 (PMC13613234; doi:10.1093/plcell/koag264)
Supplement: Supplementary file 1 [file koag264_supplementary_data.zip › 20260818_HEAL_2026_supplementary_figures_and_tables.pdf]

Diverse haplotypes at a complex *Solanum americanum* locus confer resistance to *Phytophthora infestans* and *P. capsici*

Robert Heal[1], Andrea Carolina Olave-Achury[1,3], Maria Sindalovskaya[1,4], Ruth Norkor Annang[1], Shuting Sun[1], He Zhao[1], Qing Liu[2], Li Long[1], Hari Sharan Karki[1], Jodie Taylor[1], Aleksandra Wawryk-Khamdavong[1], Kinga Bachowska[1], Matthew Smoker[1], Maheen Alam[1], Sarah Pottinger[1], Vatsal Arora[1], Kee Hoon Sohn[5,6,7,8,9], Kamil Witek[1,10,*], Xiao Lin[2,1,11,*], and Jonathan D G Jones[1,*]

Addresses:

1. The Sainsbury Laboratory, University of East Anglia, Norwich Research Park, Colney Lane, Norwich, NR4 7UH, UK.
2. Department of Agri-microbiomics and Biotechnology, State Key Laboratory of Microbial Diversity and Innovative Utilization, Institute of Microbiology, Chinese Academy of Sciences, Beijing 100101, China
3. Earlham Institute, Norwich Research Park, Norwich, NR4 7UZ, UK.
4. John Innes Centre, Norwich Research Park, Colney Lane, Norwich, NR4 7UH, UK.
5. Department of Agricultural Biotechnology, Seoul National University, Seoul 08826, Republic of Korea
6. Global Plant Immunity Research Center, Seoul National University, Seoul 08826, Republic of Korea
7. Research Institute of Agriculture and Life Sciences, Seoul National University, Seoul 08826, Republic of Korea
8. Plant Genomics and Breeding Institute, Seoul National University, Seoul 08826, Republic of Korea
9. Plant Health Center, Seoul National University, Seoul 08826, Republic of Korea
10. 2Blades, Evanston, IL, 60201, USA
11. University of the Chinese Academy of Sciences, Beijing 100049, China

* Co-corresponding author

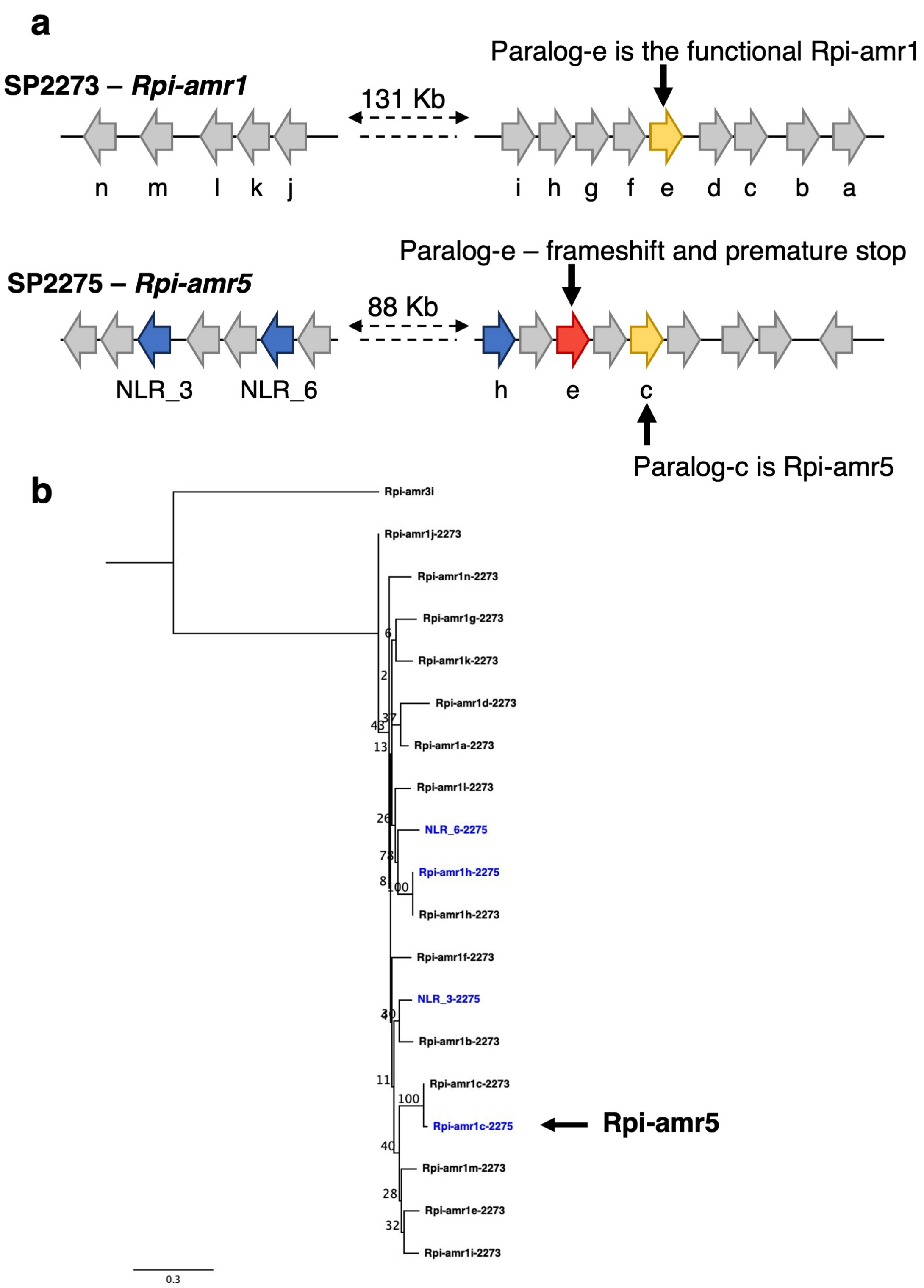

Supplementary figure 1. Alignment to NLRs from the SP2273 *Rpi-amr1* cluster was used to name *Rpi-amr5* candidates. (a) The *Rpi-amr1* clusters in *S. americanum* accessions SP2273 (the original source of *Rpi-amr1*) and SP2275 (the source of *Rpi-amr5*). NLR-encoding genes are represented using arrows, genes which confer *P. infestans* resistance are coloured yellow, other *Rpi-amr5* candidates are coloured blue. The *Rpi-amr1* ortholog (*Rpi-amr1e*, indicated in red) is non-functional in SP2275 due to a premature stop codon. **(b)** Phylogenetic tree showing the relationship of the Rpi-amr5 candidates with NLRs of the Rpi-amr1 cluster from the accession SP2273. This tree was used to name two candidates which have close homology to SP2273 NLRs, these are Rpi-amr1c-2275 (the functional Rpi-amr5) and Rpi-amr1h-2275. The other two candidates were generically named NLR_3-2275 and NLR_6-2275 based on their position within the SP2275 cluster. The functional Rpi-amr5 is indicated. The tree is rooted using Rpi-amr3.

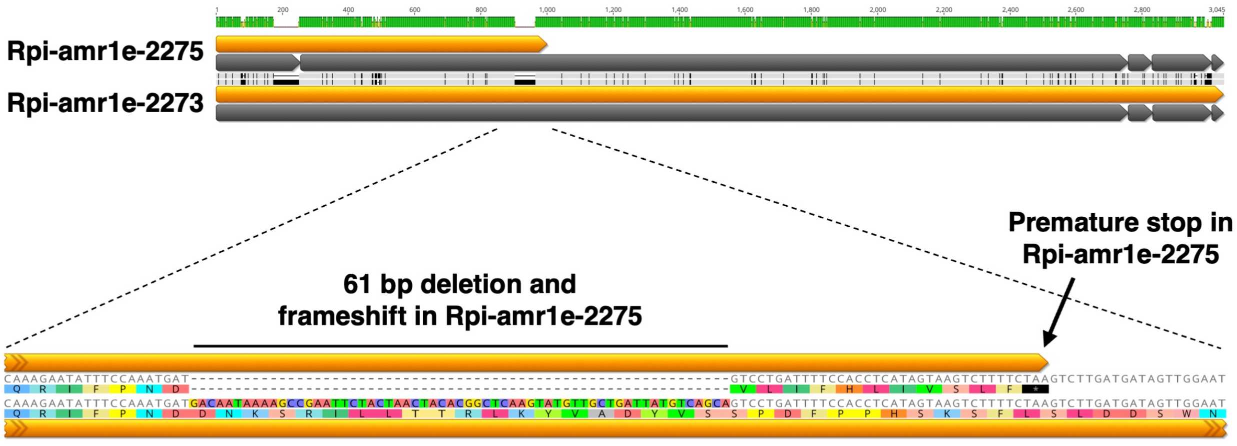

Supplementary figure 2. Comparison of *Rpi-amr1e-2275* to the functional *Rpi-amr1-2273* demonstrates that resistance in SP2275 is not conferred by *Rpi-amr1*. Resistance in SP2275 is not due to *Rpi-amr1*. The *Rpi-amr1e* paralog, which is responsible for resistance in SP2273, is non-functional in SP2275 due to a frameshift and premature stop. The position of this stop is indicated in the alignment. The CDS of each allele is shown, grey bars indicate the position of exons and orange bars the ORF.

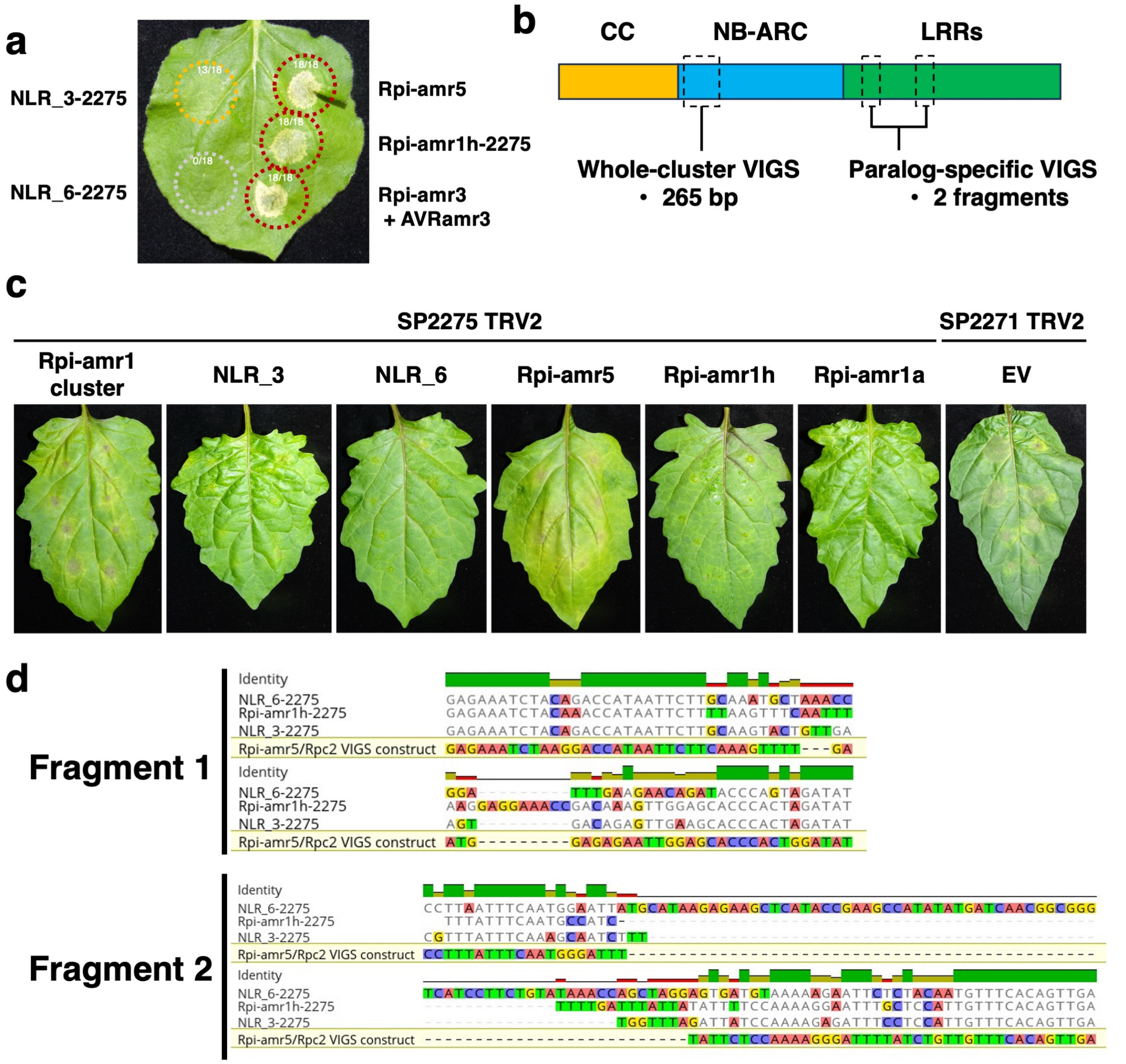

e

		NLR_3-2275	NLR_6-2275	Rpi-amr1h-2275
Fragment 1	Percent identity	78.3%	56.5%	67.9%
	>80% 21-mer hits	0	0	0
Fragment 2	Percent identity	69.6%	32.6%	67.6%
	>80% 21-mer hits	0	0	0

Supplementary figure 3. VIGS of *Rpi-amr5* compromises resistance to *P. infestans* in SP2275. (a) Transient expression of *Rpi-amr5* and *Rpi-amr1h-2275* in *N. benthamiana* results in strong HR indicating constitutive activity. *N. benthamiana* infiltrations were performed using *Agrobacterium* suspensions at 0.2 OD_{600}, leaves were imaged at 4 dpi. **(b)** VIGS was used to test the functionality of *Rpi-amr5* candidates. Whole-cluster silencing was performed using a fragment from the NB-ARC encoding region of the *Rpi-amr1h* paralog. Paralog-specific silencing was performed using constructs containing two LRR-encoding fragments fused together. **(c)** Silencing of the *Rpi-amr1* cluster and *Rpi-amr5* compromised resistance in SP2275, silencing of *Rpi-amr1a* or *Rpi-amr1h* did not affect resistance. Leaves were inoculated with 10 µl drops of *P. infestans* (strain 88069) spores at a concentration of 20,000 spores mL^{-1} and photographed at 5 days post-inoculation. The absence of off-target silencing was not experimentally validated; therefore, it remains possible that additional paralogs also function in *P. infestans* recognition. **(d)** Alignment of the *Rpi-amr5*-specific VIGS construct with other candidate NLRs. Sequences shown were extracted from an alignment of the CDS sequences of *Rpi-amr5* candidates (as defined in Supplementary Table 1). **(e)** Table showing the percentage nucleotide identity of each *Rpi-amr5* candidate within the VIGS region that compromises *Rpi-amr5*-mediated resistance in *S. americanum* accession SP2275. The number of predicted off-target hits (21-mer sequences with >80% identity to other sequences) is also indicated.

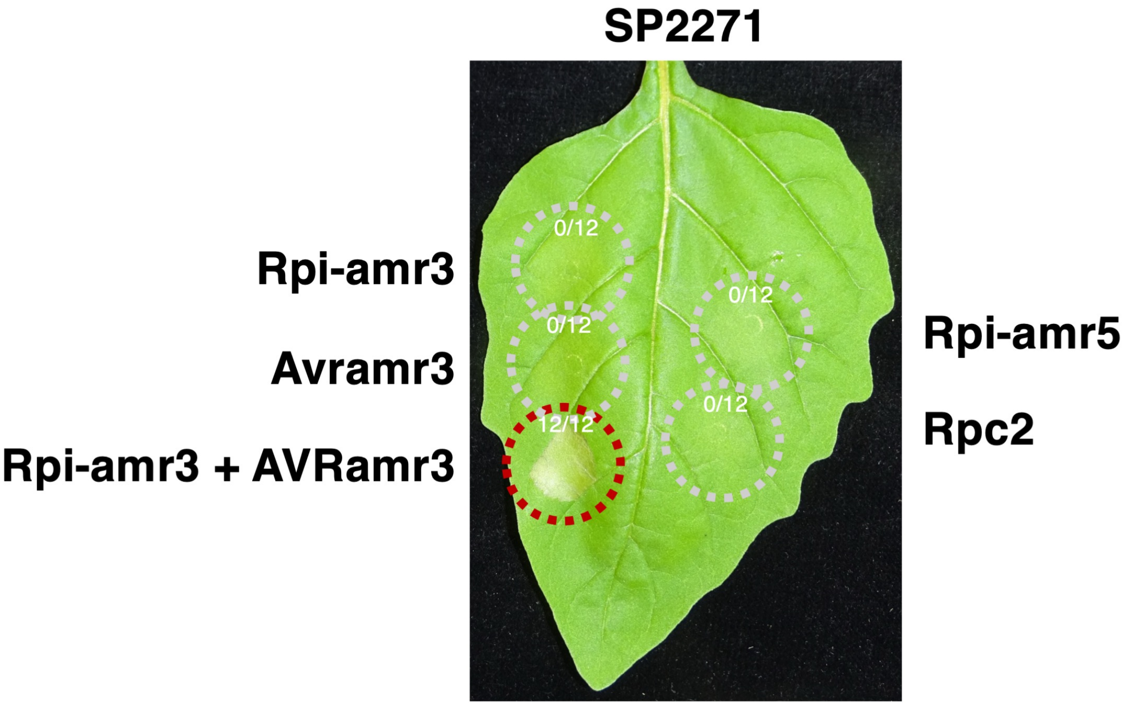

Supplementary figure 4. Transient expression of *Rpi-amr5* and *Rpc2* does not result in constitutive activity in *S. americanum*. *Agrobacterium*-mediated transient expression of *Rpi-amr5* and *Rpc2* does not result in HR in *S. americanum* accession SP2271. *S. americanum* infiltrations were performed at 0.5 OD_{600}, leaves were imaged at 3 dpi.

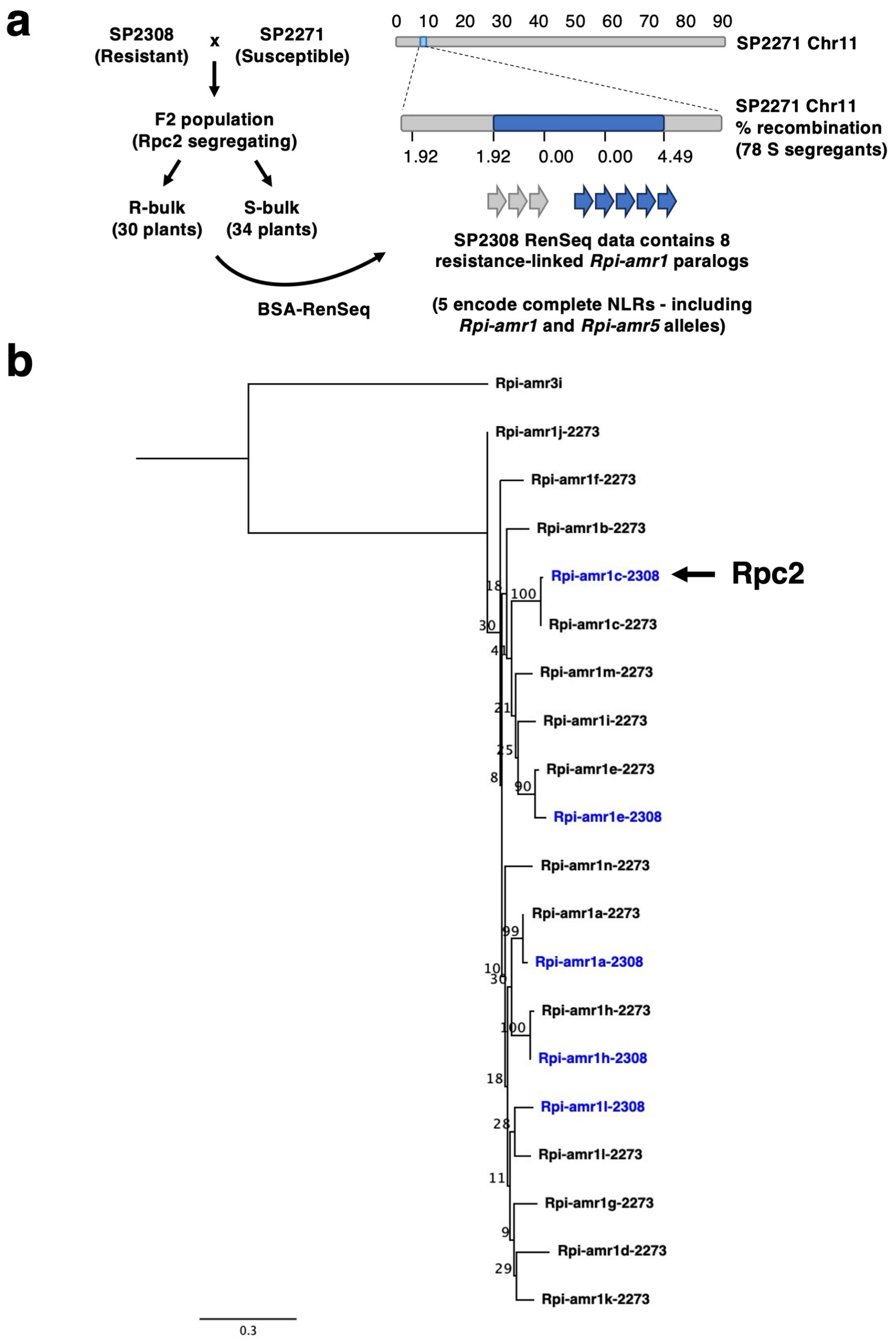

Supplementary figure 5. Alignment to NLRs from the SP2273 *Rpi-amr1* cluster was used to name *Rpc2* candidates. (a) SP2308 was crossed to SP2271 and an F2 population was phenotyped, the 34 most susceptible and 30 most resistant plants were used for bulked segregant analysis. Using the SP2271 reference genome, *Rpc2* was mapped to the *Rpi-amr1* cluster on Chromosome 11. Fine mapping was performed using an expanded population of 78 susceptible segregants, percentage recombination rates are indicated. Eight NLR-encoding genes within this interval were identified by comparing SP2308 SMRT RenSeq contigs with genes from the corresponding region of the SP2273 Rpi-amr1 cluster. The five complete candidate genes are indicated in blue. Both *Rpi-amr1* and *Rpi-amr5* are present as complete genes in SP2308. **(b)** Phylogenetic tree showing the relationship between Rpc2 candidates and NLRs from the *Rpi-amr1* cluster in *S. americanum* accession SP2273. This tree was used to name the candidates – Rpi-amr1a-2308, Rpi-amr1c-2308 (the functional Rpc2), Rpi-amr1e-2308, Rpi-amr1h-2308 and Rpi-amr1l-2308. The functional Rpc2 is indicated. The tree is rooted using Rpi-amr3.

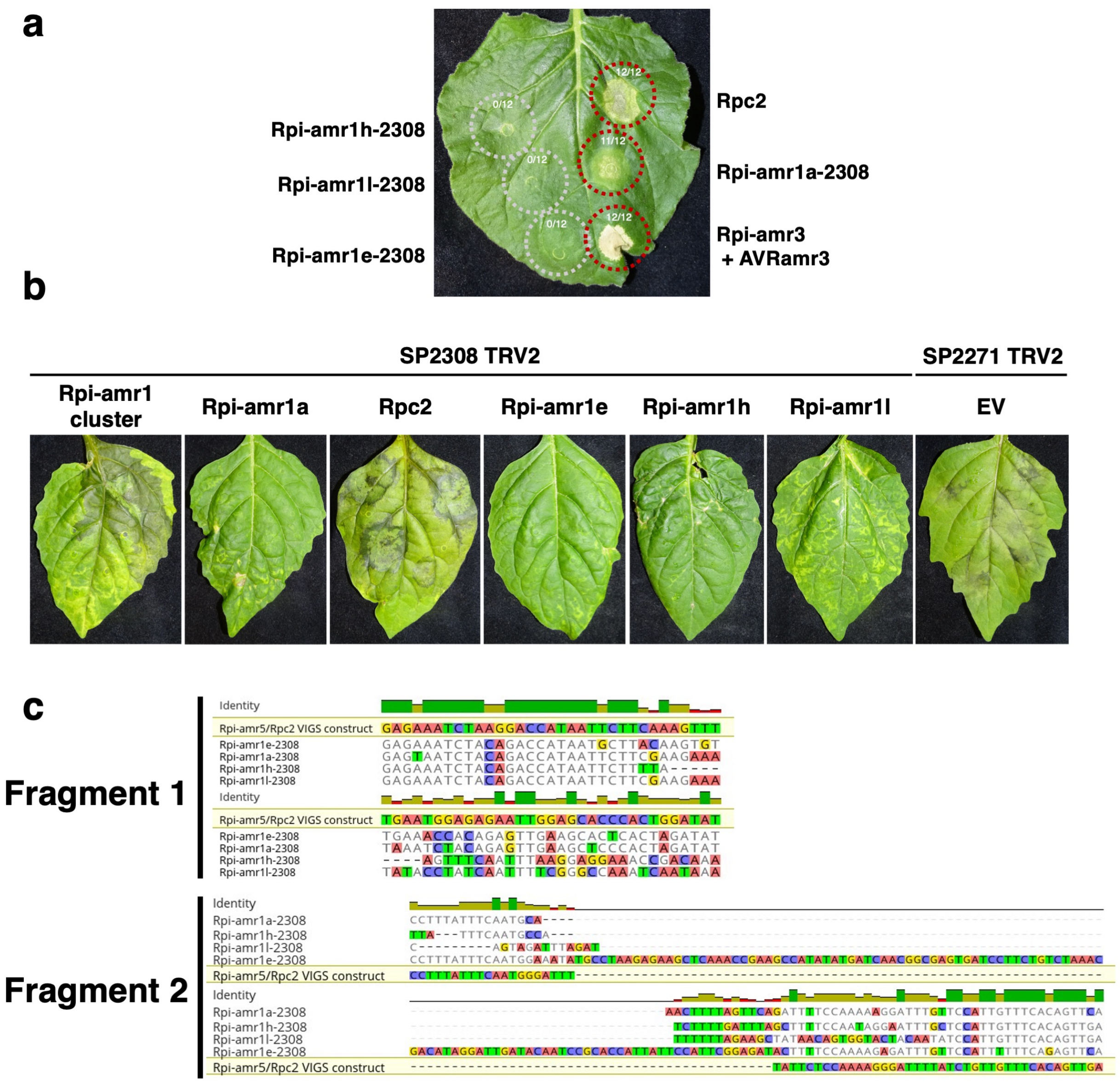

d

		NLR_1	NLR_2	NLR_3	NLR_7
**Fragment 1**	Percent identity	50%	56.1%	78.8%	77.3%
	>80% 21-mer hits	0	0	0	0
**Fragment 2**	Percent identity	55.6%	38.7%	27.4%	63%
	>80% 21-mer hits	0	0	0	0

Supplementary figure 6. VIGS of the *Rpc2* compromises *P. capsici* resistance in SP2308. (a) *Rpc2* and the four complete paralogs within the SP2308 *Rpi-amr1* cluster were cloned into CaMV *35S* expression vectors for testing in *N. benthamiana*. Transient expression of either *Rpc2* or *Rpi-amr1a-2308* produced strong HR, indicating constitutive activity. *N. benthamiana* infiltrations were performed at 0.2 OD_{600}, leaves were imaged at 4 dpi. **(b)** VIGS was performed to silence candidates in SP2308. Silencing of either the whole cluster, or *Rpc2* compromised resistance in SP2308. Silencing of *Rpi-amr1e-2308* did not result in susceptibility. Leaves were inoculated with 10 µl drops of *P. capsici* strain LT1534 at a concentration of 20,000 spores ml^{-1} and imaged at 3 days post-inoculation. The absence of off-target silencing was not experimentally validated; therefore, it remains possible that additional paralogs may also function in *P. capsici* recognition. **(c)** Alignment of the *Rpc2*-specific VIGS construct with other candidate NLRs within the *Rpc2* cluster (as defined in Supplementary Table 2). Sequences shown were extracted from an alignment of the CDS sequences of *Rpc2* candidates. **(d)** Table showing the percentage nucleotide identity of each *Rpc2* candidate within the VIGS region that compromises *Rpc2*-mediated resistance in *S. americanum* accession SP2308. The number of predicted off-target hits (21-mer sequences with >80% identity to other sequences) is also indicated.

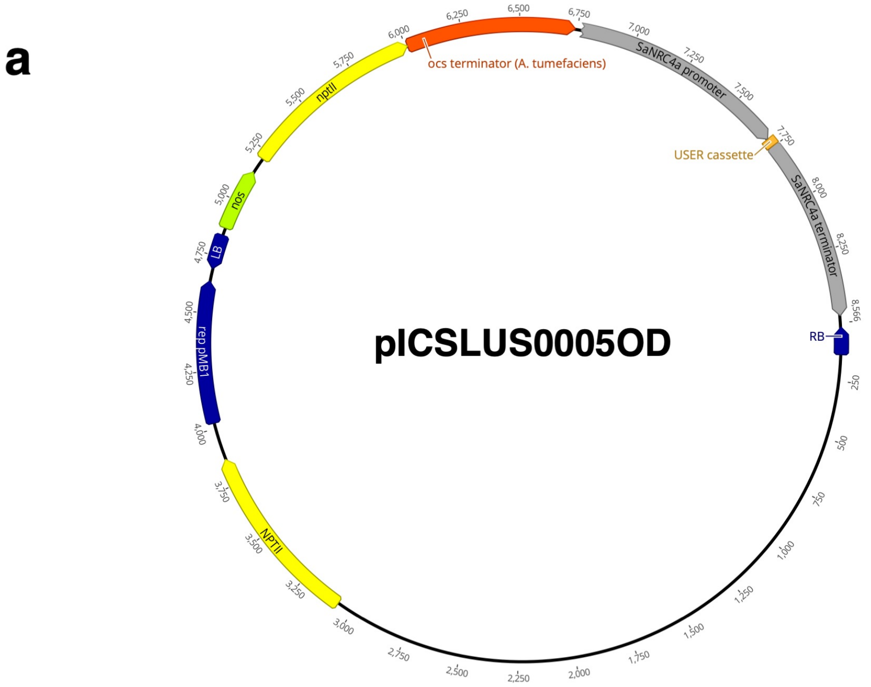

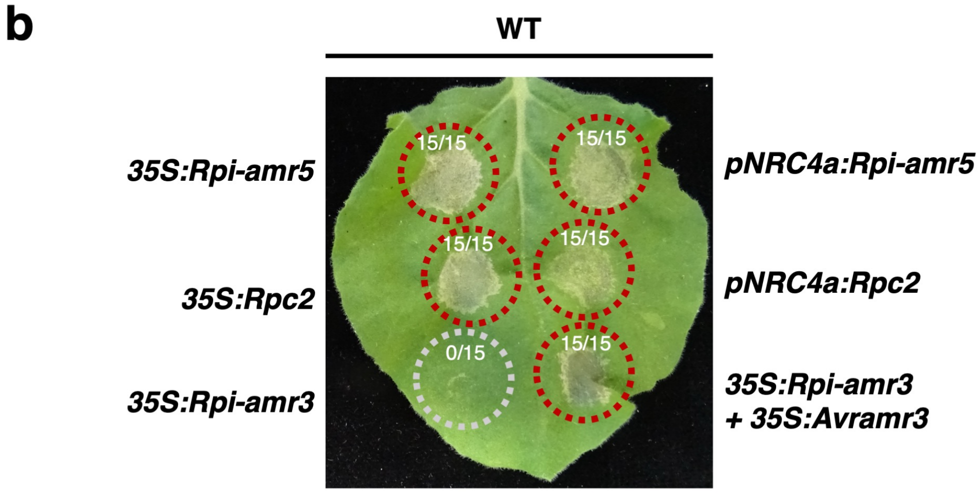

Supplementary figure 7. Constitutive activity of Rpi-amr5 and Rpc2 in *N. benthamiana* is observed following expression under an NLR promoter. (a) Custom USER vector pICSLUS0005OD was designed to express NLRs under a moderate promoter. The *SaNRC4a* promoter (943 bp) and terminator (793 bp) was cloned from the *S. americanum* reference accession SP1102. A USER cassette was fused between these sequences to facilitate linearisation of the plasmid and insertion of target sequences. **(b)** *Rpi-amr5* and *Rpc2* confer constitutive activity in *N. benthamiana* under both CaMV *35S* and *S. americanum NRC4a* regulatory elements. Clear cell death was visible after 3 days. Cell death is equivalent to effector-dependent activation of Rpi-amr3. *Agrobacterium* suspensions were infiltrated at 0.2 OD_{600}.

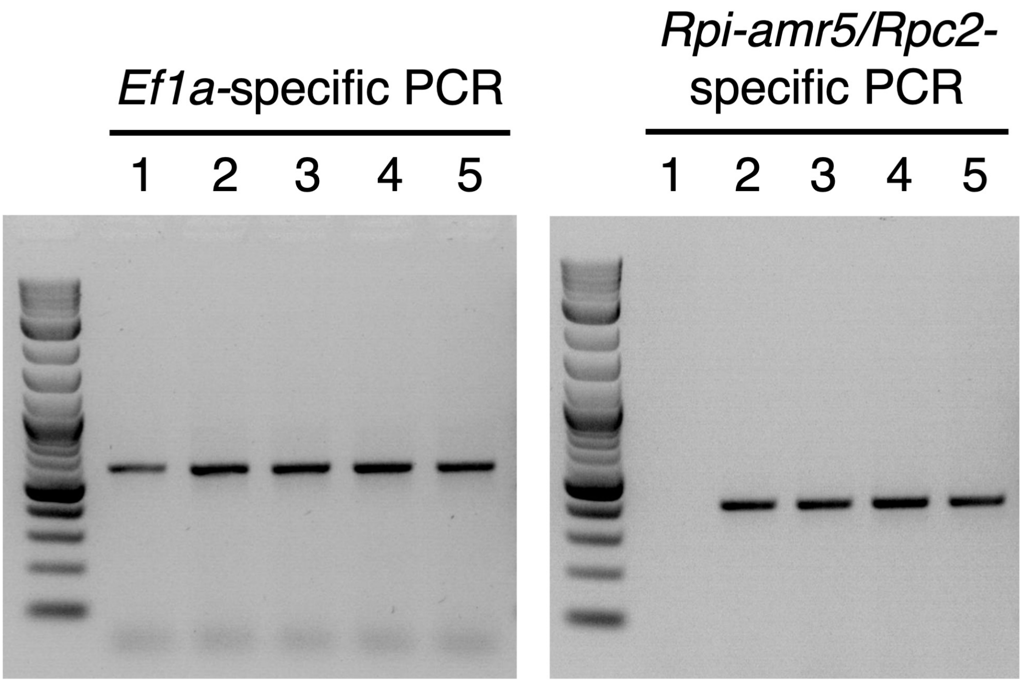

1 – Untransformed *S. lycopersicum* (Moneymaker)
2 – 35S:Rpc2 line 1 (Moneymaker background)
3 – 35S:Rpc2 line 2 (Moneymaker background)
4 – 35S:Rpi-amr5 line 1 (Moneymaker background)
5 – 35S:Rpi-amr5 line 2 (Moneymaker background)

Supplementary figure 8. RT-PCR was used to confirm expression of transgenes in *S. lycopersicum* lines. To confirm the expression of *Rpi-amr5* and *Rpc2* in transgenic *S. lycopersicum* lines, RT-PCR was performed. cDNA was synthesised from RNA extracted from leaf tissue of mature plants. Transgene expression was assessed using a primer pair that anneals to either *Rpi-amr5* or *Rpc2*. A control PCR amplifying *EF1a* was used to confirm successful cDNA synthesis.

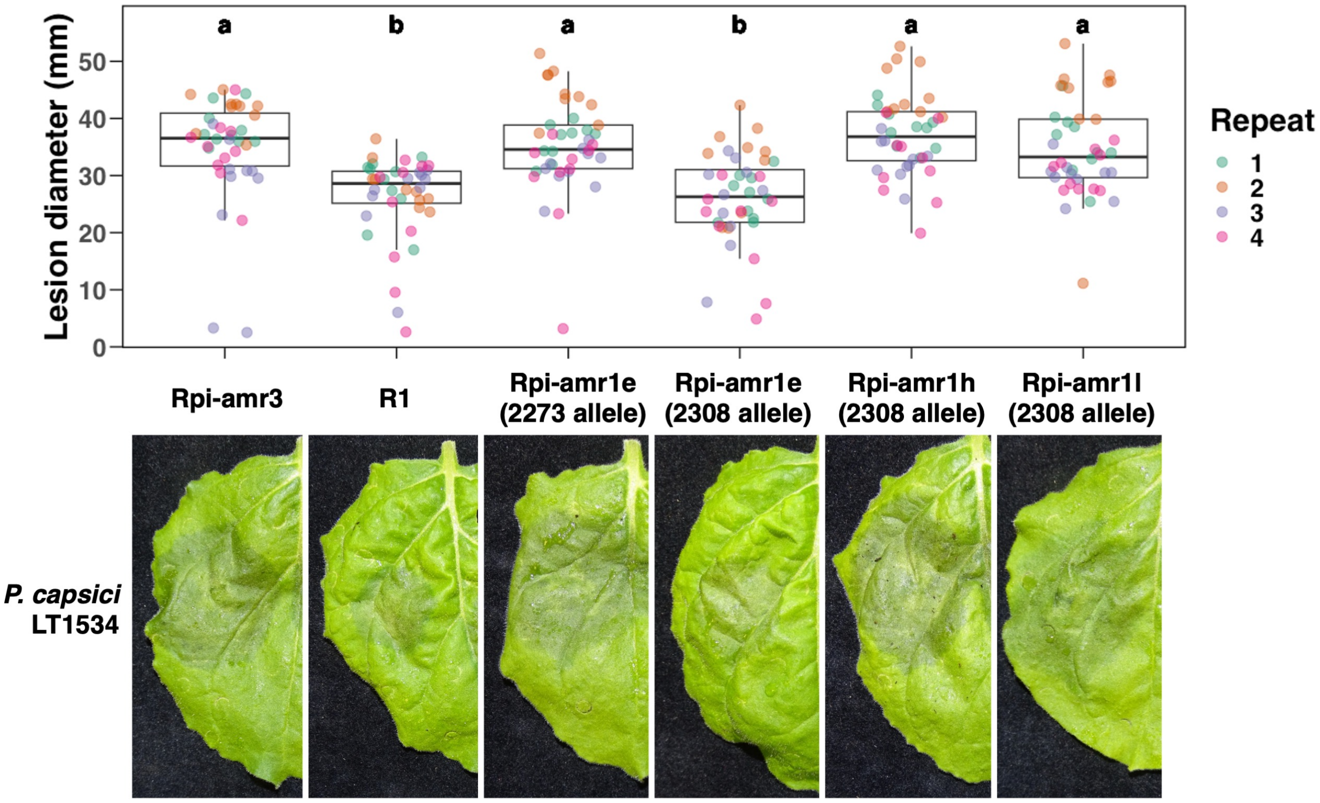

Supplementary figure 9. Transient expression of *Rpi-amr1e-2308*, but not other paralogs, results in elevated resistance to *P. capsici* in *N. benthamiana*. *Rpi-amr1e-2308* confers partial resistance to *P. capsici* in *N. benthamiana* transient assays. Other non-constitutively active paralogs from the *Rpi-amr1* cluster in SP2308 do not. *R1* was used as a positive control for *P. capsici* resistance. Agroinfiltration was performed using suspensions normalised to 0.5 OD_{600}. At 2 dpi, leaves were detached and *P. capsici* mycelial plugs were transferred onto the leaves. Lesion size was measured at 3 days post-inoculation. Four biological replicates were performed, all data points (40 per treatment) are represented as box and whisker plots. Statistical differences determined using one-way ANOVA and Tukey's HSD test ($p < 0.05$).

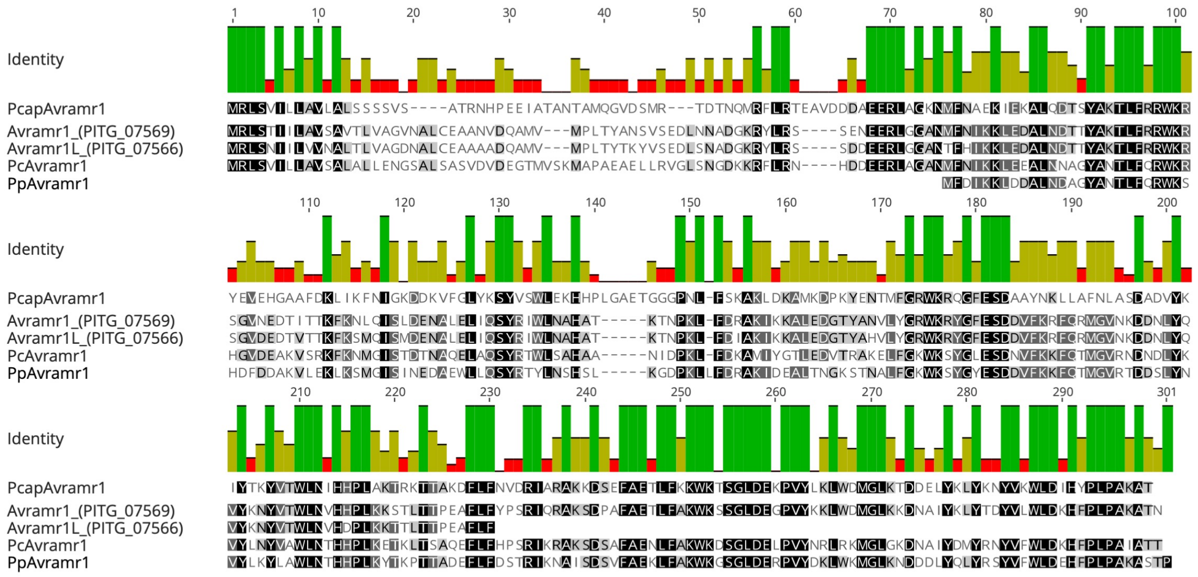

Supplementary figure 10. AVRamr1 amino acid alignment highlights the divergence of effector orthologs recognised by Rpi-amr1. The alignment of AVRamr1 protein sequences from *Phytophthora infestans* (Pi), *Phytophthora cactorum* (Pc), *Phytophthora parasitica* (Pp) and *Phytophthora capsici* (Pcap). PcapAVRamr1 was identified in this study, other alleles were identified by Lin et al. 2020.

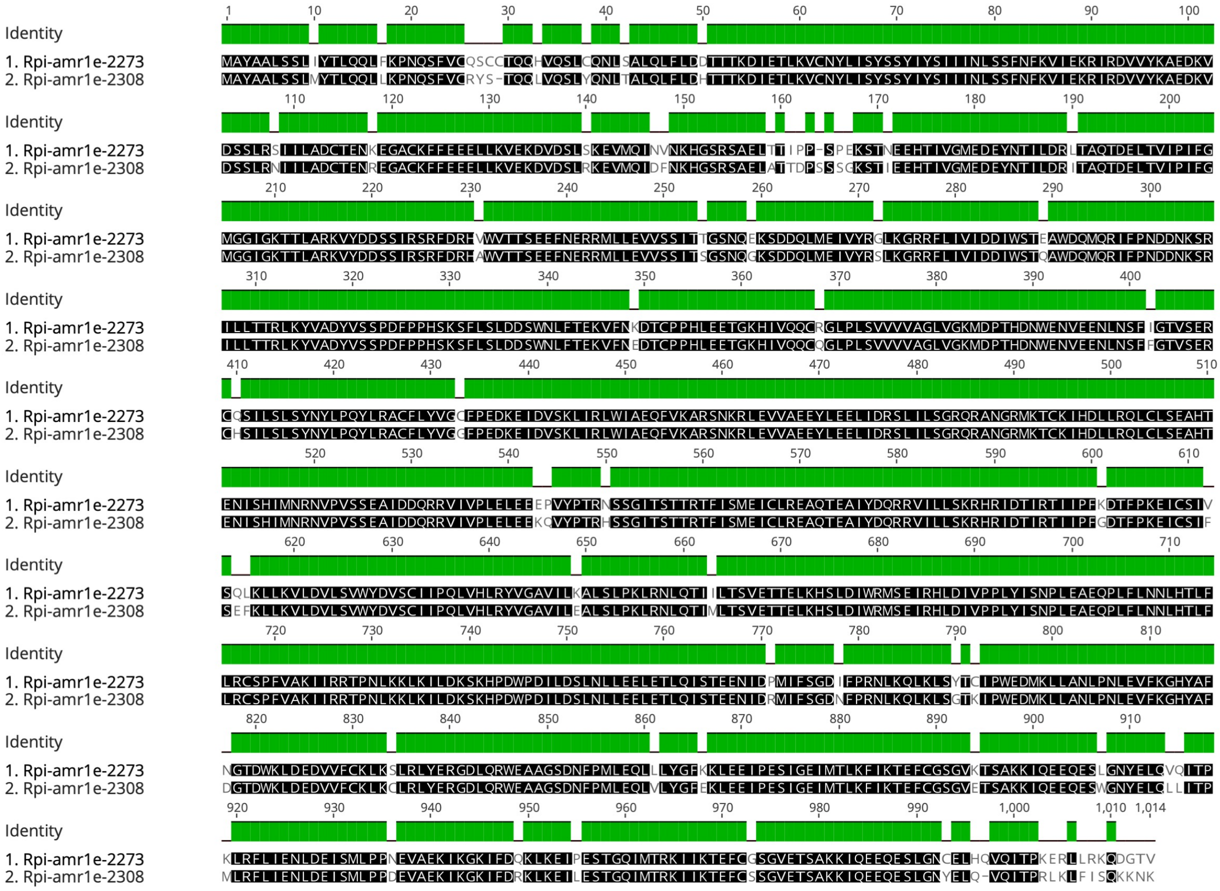

Supplementary figure 11. Alignment of Rpi-amr1-2273 and Rpi-amr1-2308 demonstrates that polymorphisms are distributed throughout the protein sequences. The protein alignment of Rpi-amr1 sequences from *S. americanum* accessions SP2273 and SP2308. The SP2273 allele was characterised by Witek et al 2021, the SP2308 allele was cloned in this study. The *Rpi-amr1-2308* gene model was predicted through homology to the published *Rpi-amr1-2273* model.

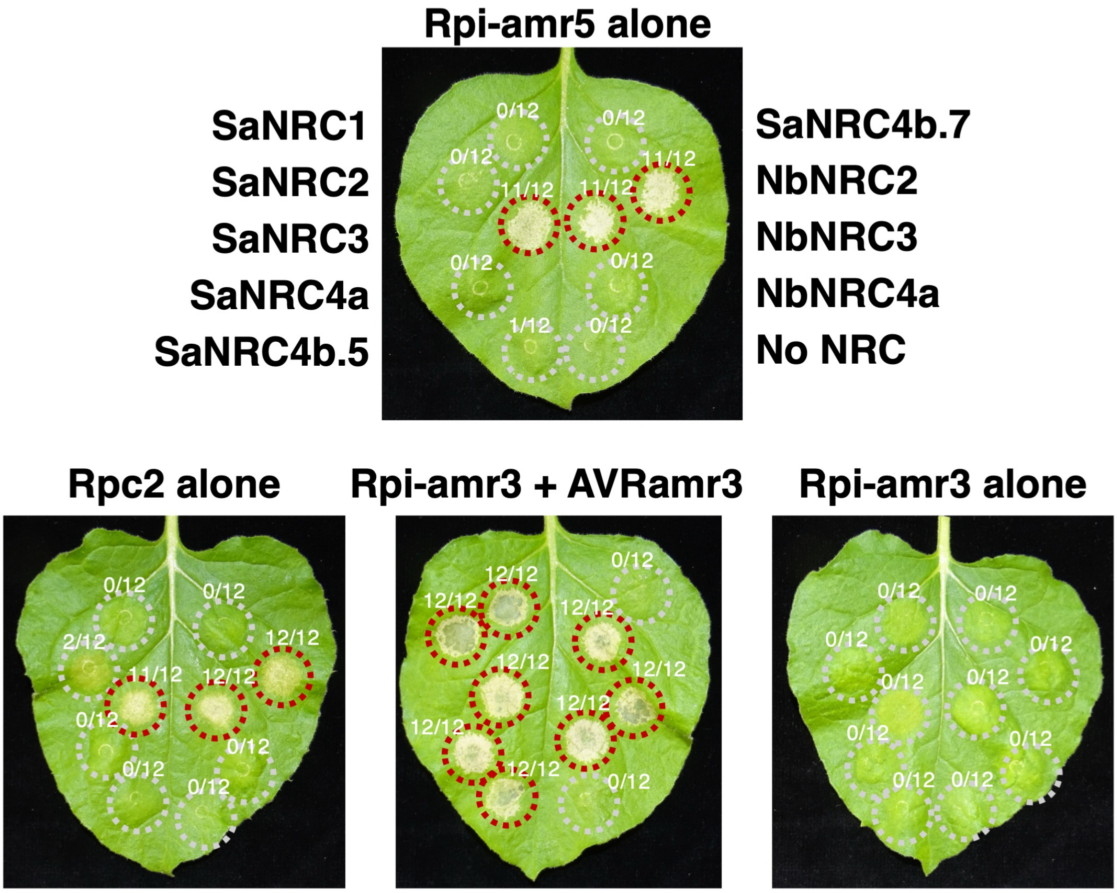

Supplementary figure 12. Rpi-amr5 and Rpc2 are constitutively active when expressed with NbNRC2, NbNRC3, or SaNRC3, but not SaNRC2. Absence of NRC helper NLRs abolishes Rpi-amr5/Rpc2 constitutive activity. Complementation with NbNRC2, NbNRC3, or SaNRC3 restores constitutive activity. Transient expression was performed in the nrc2/3/4 knock out *N. benthamiana* line. *Agrobacterium* strains were infiltrated at 0.2 OD_{600} and leaves were imaged at 3 dpi.

a

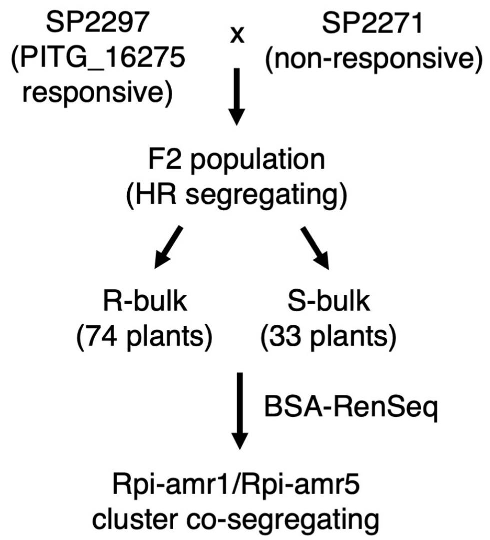

b

	Rpi-amr1e-...	Rpc2	Rpi-amr5-2...	Rpi-amr5-2...
Rpi-amr1e-2273		74.0%	74.1%	74.1%
Rpc2	74.0%		97.4%	97.4%
Rpi-amr5-2275	74.1%	97.4%		100%
Rpi-amr5-2297	74.1%	97.4%	100%	

Supplemental figure 13. *Rpi-amr5* function has been validated in three *S. americanum* accessions. (a) Recognition of PITG_16275 was mapped in an F2 population produced by crossing the responsive line SP2297 with the non-responsive line SP2271. Responsiveness segregates in a 3:1 (responsive: non-responsive) segregation ratio (121 R and 33 NR; $\chi^2(1) = 1.047$, $P = 0.3061$). Bulked segregant analysis was used to map the recognition to the *Rpi-amr1*/*Rpi-amr5*/*Rpc2* cluster on Chromosome 11. **(b)** Functionally validated alleles encode NLRs with high amino acid identity. Heatmap of the percent identity of aligned amino acid sequence of these alleles. All three were independently mapped using either resistance to *P. infestans* (*Rpi-amr5-2275*), resistance to *P. capsici* (*Rpc2*), or recognition of PITG_16275 (*Rpi-amr5-2297*).

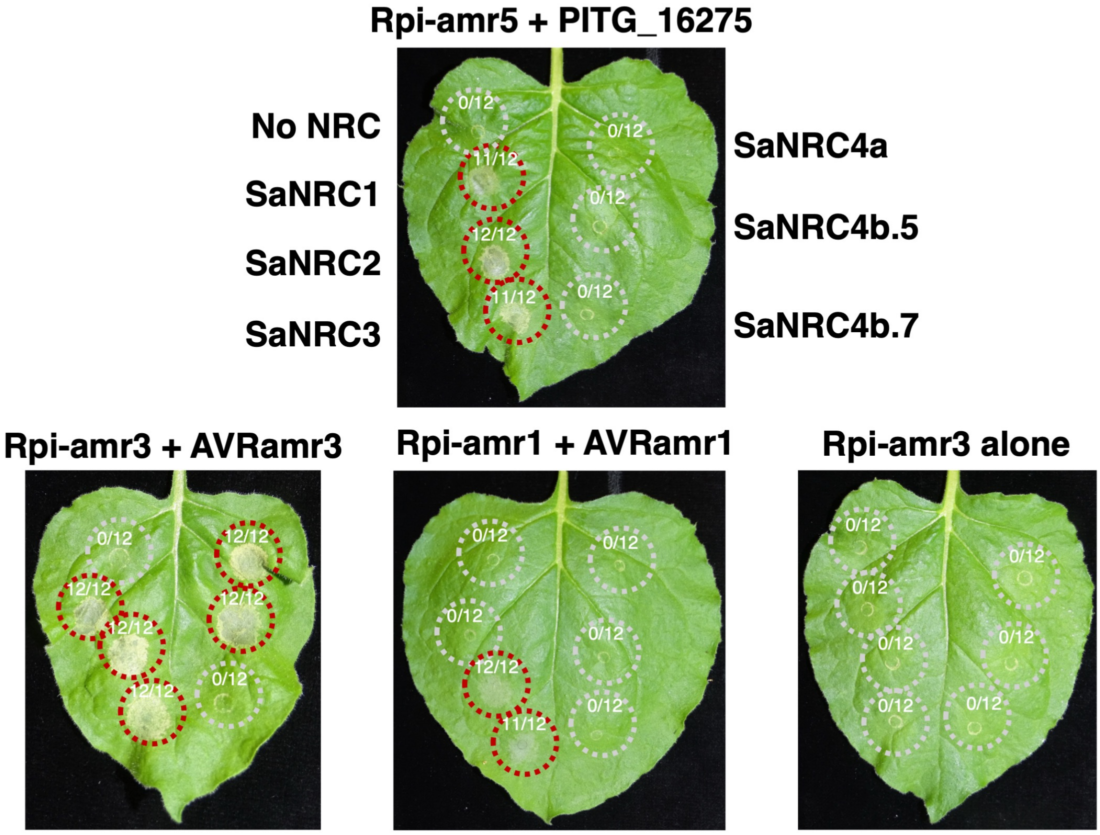

Supplementary figure 14. Co-infiltration in *N. benthamiana* nrc2/3/4 lines reveal that Rpi-amr5 and Rpc2 can signal through either SaNRC1 or SaNRC2. PITG_16275-mediated activation of Rpi-amr5 and Rpc2 triggers SaNRC1- and SaNRC2- dependent cell death. SaNRC3 mediated cell death cannot be distinguished from effector-independent constitutive activity (demonstrated in Fig. S14). Transient expression was performed in the nrc2/3/4 knock out *N. benthamiana* line. *Agrobacterium* strains were infiltrated at 0.2 OD_{600} and leaves were imaged at 3 dpi.

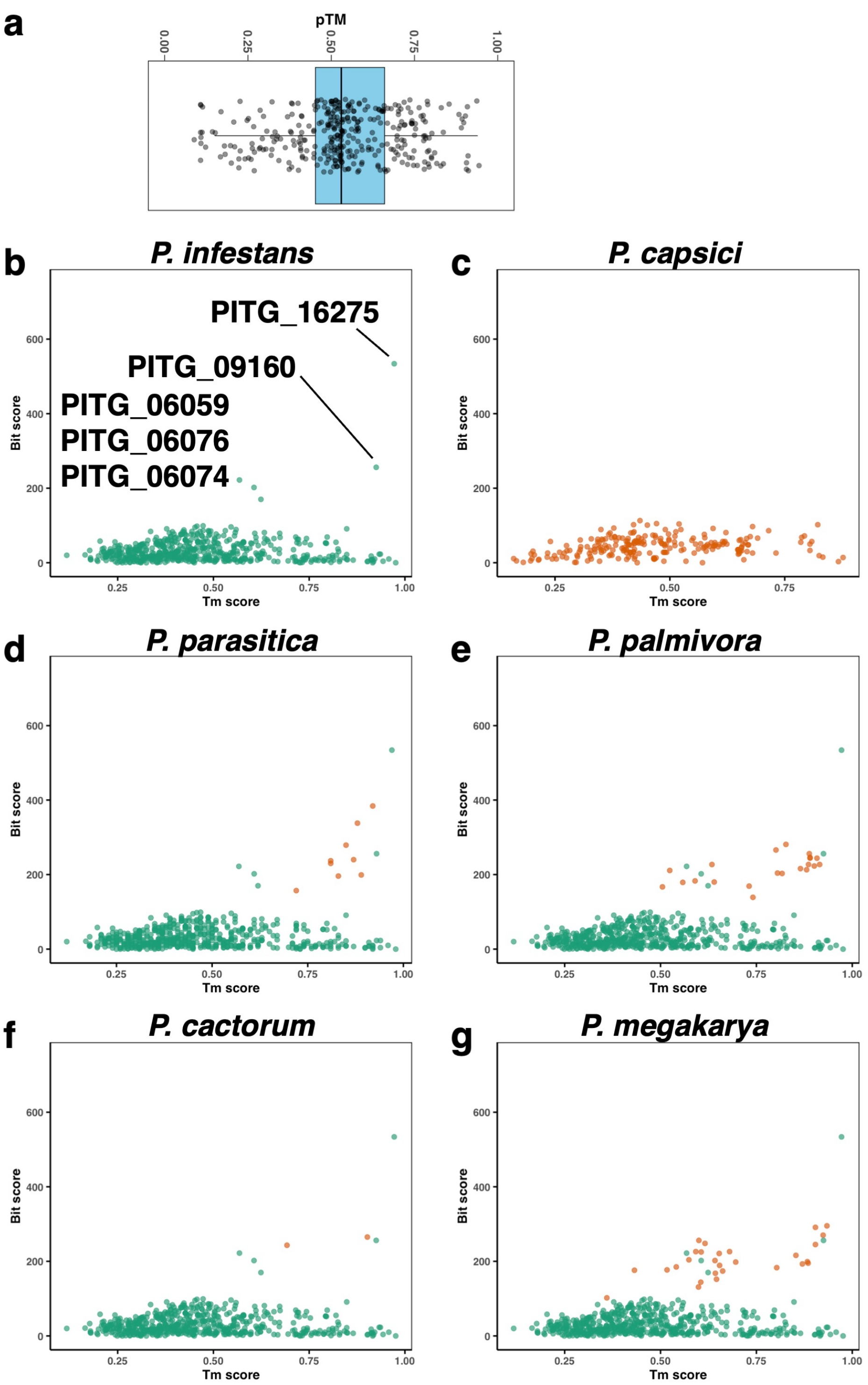

Supplemental figure 15. Foldseek analysis demonstrates that structural homologs of PITG_16275 are present in several *Phytophthora* species, but not in *P. capsici*. (a) AlphaFold3 was used to predict the structures of the RXLR effectors predicted from *P. capsici*. The highest-ranking model for each effector was chosen, a distribution of pTM scores for these effectors is shown. **(b)** *P. infestans* contains four additional effectors with predicted structural similarity. A Foldseek search was performed using a local database of *P. infestans* effector models (generated by Seong and Krasileva, 2023), a new model of PITG_16275 was used as an input. Each effector is represented by a data point in the plot. The hits with high Bit score and Tm score were inspected as possible structural homologs. **(c)** No *P. capsici* effector structures were identified with similarity to PITG_16275. A Folkseek search was performed using a structure of PITG_16275 predicted by AlphaFold3, against a library of *P. capsici* effectors whose structures were predicted using AlphaFold3. **(d)** Unlike *P. capsici*, structural homologs of PITG_16275 were identified in *P. parasitica*, as well as *P. palmivora* **(e)**, *P. cactorum* **(f)** and *P. megakarya* **(g)**. These hits are indicated in orange, their position within the plot from panel B is shown.

a

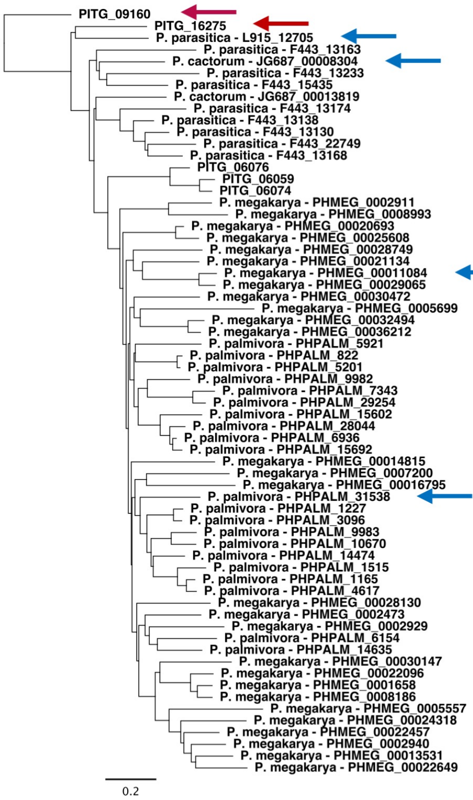

b

	PITG_09160	P. cactorum	P. megakar...	P. palmivora	PITG_16275	P. parasitica
PITG_09160		29.9%	29.0%	29.9%	38.8%	40.8%
P. cactorum	29.9%		43.8%	38.5%	45.5%	43.4%
P. megakarya	29.0%	43.8%		51.0%	48.5%	50.0%
P. palmivora	29.9%	38.5%	51.0%		42.4%	51.5%
PITG_16275	38.8%	45.5%	48.5%	42.4%		60.0%
P. parasitica	40.8%	43.4%	50.0%	51.5%	60.0%	

c

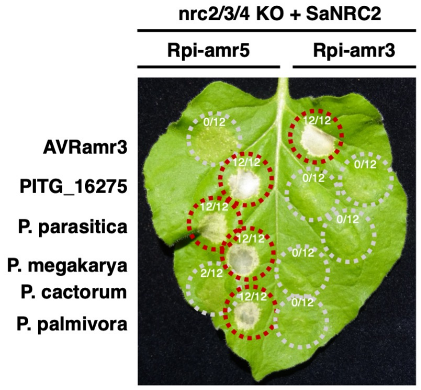

Supplementary figure 16. PITG_16275 orthologs from *P. parasitica*, *P. megakarya*, and *P. palmivora* have low sequence identity to the *P. infestans* effector but nevertheless recognised by Rpi-amr5. (a) Orthologs of PITG_09160 and PITG_16275 are found in *P. megakarya* (28), *P. palmivora* (21), *P. parasitica* (8), and *P. cactorum* (2). Orthologs were identified using NCBI BLAST and local BLAST performed on the *Phytophthora* reference genomes. PITG_09160 and PITG_16275 are indicated with red arrows. Cloned orthologs from *P. megakarya*, *P. palmivora*, *P. parasitica,* and *P. cactorum* are indicated by blue arrows, these were selected as the highest scoring hits from the Foldseek search. **(b)** Heatmap of the recognised *P. infestans* effectors with the cloned orthologs from *P. megakarya*, *P. palmivora*, *P. parasitica* and *P. cactorum*. **(c)** Rpi-amr5 can recognise PITG_16275 orthologs from *P. megakarya*, *P. palmivora,* and *P. parasitica.* No HR was observed upon co-expression with the cloned *P. cactorum* ortholog. Effectors were co-expressed with sensor NLRs and SaNRC2 in the *N. benthamiana nrc2/3/4* KO line. *Agrobacterium* strains infiltrated at 0.3 OD_{600}, images were taken at 3 dpi.

a

	P.cap_E144	P.cap_E149	P.cap_E150	PITG_09160	PITG_16275
P.cap_E144		65.4%	65.6%	10.4%	15.2%
P.cap_E149	65.4%		92.5%	11.8%	15.9%
P.cap_E150	65.6%	92.5%		11.8%	15.2%
PITG_09160	10.4%	11.8%	11.8%		39.2%
PITG_16275	15.2%	15.9%	15.2%	39.2%	

b

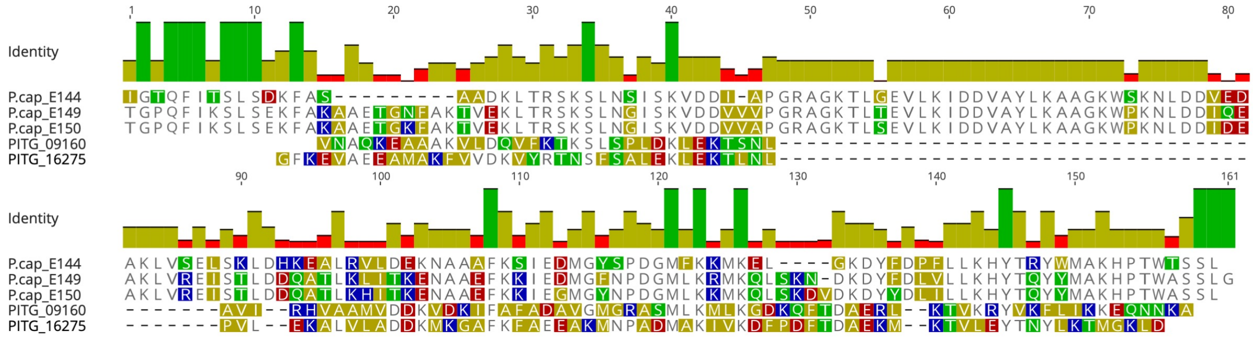

Supplementary figure 17. Protein sequence alignment shows that the *P. capsici* effectors recognised by both Rpi-amr5 and Rpc2 share low sequence similarity to PITG_16275 or PITG_09160. (a) Heatmap showing the percent identity of the post-EER amino acid sequences for the five effectors recognised by Rpi-amr5 and Rpc2. PITG_09160 and PITG_16275, from *P. infestans*, have low sequence similarity to PcE144, PcE149 and PcE150, from *P. capsici*. **(b)** Alignment of the five effectors (sequence after the EER motif) recognised by Rpi-amr5 and Rpc2.

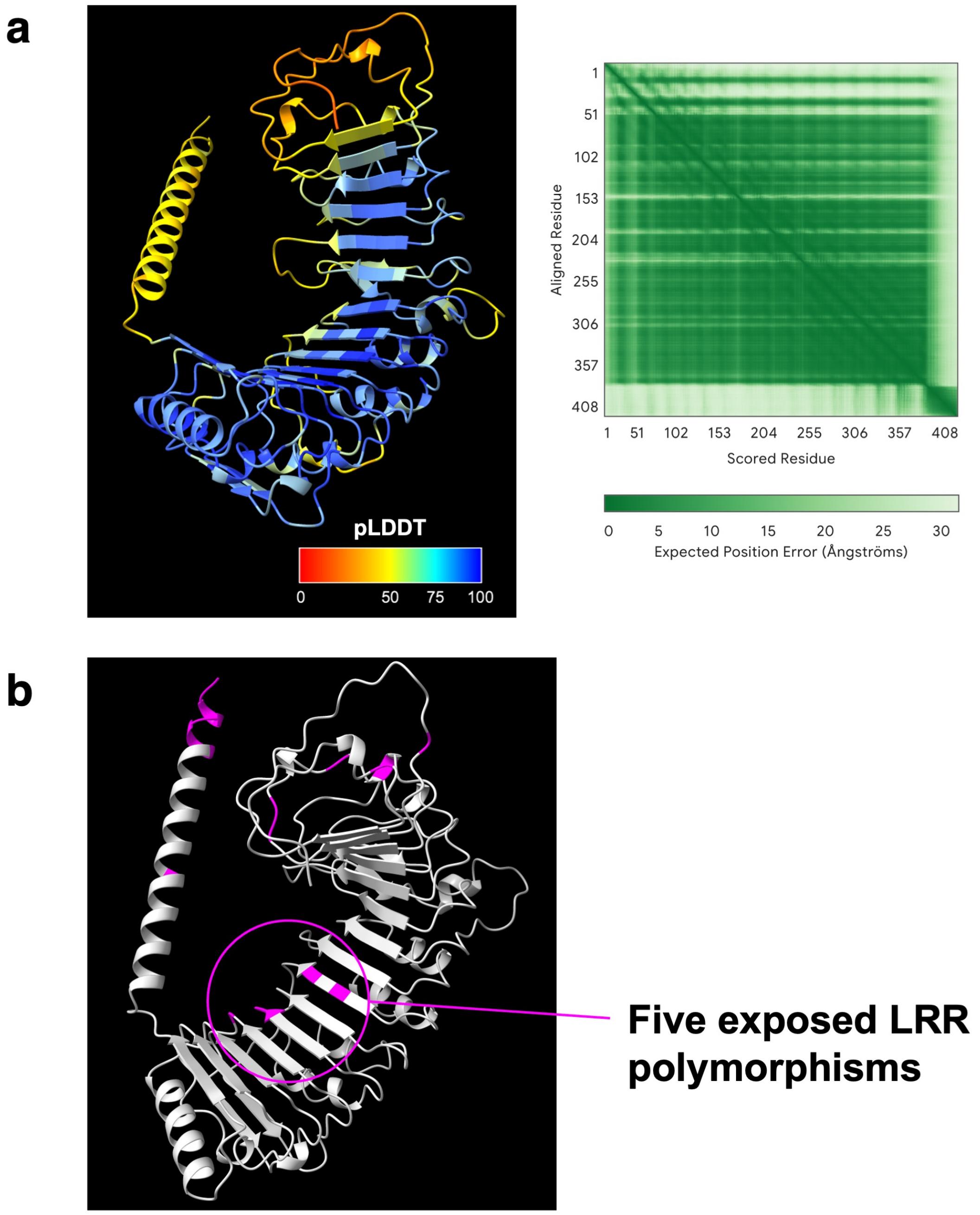

Supplementary figure 18. AlphaFold3 modelling reveals the positions of polymorphisms between Rpc2 and Rpi-amr5. (a) AlphaFold3 was used to predict the structure of the Rpc2 LRR domain. The predicted aligned error (PAE) plot and per-residue pLDDT confidence scores (indicated on the model) are also shown. **(b)** Polymorphisms between Rpc2 and Rpi-amr5 are highlighted within the LRR domain. These polymorphisms may underlie the differential recognition of PcE99 and the resistance to *P. capsici* conferred by *Rpc2*.

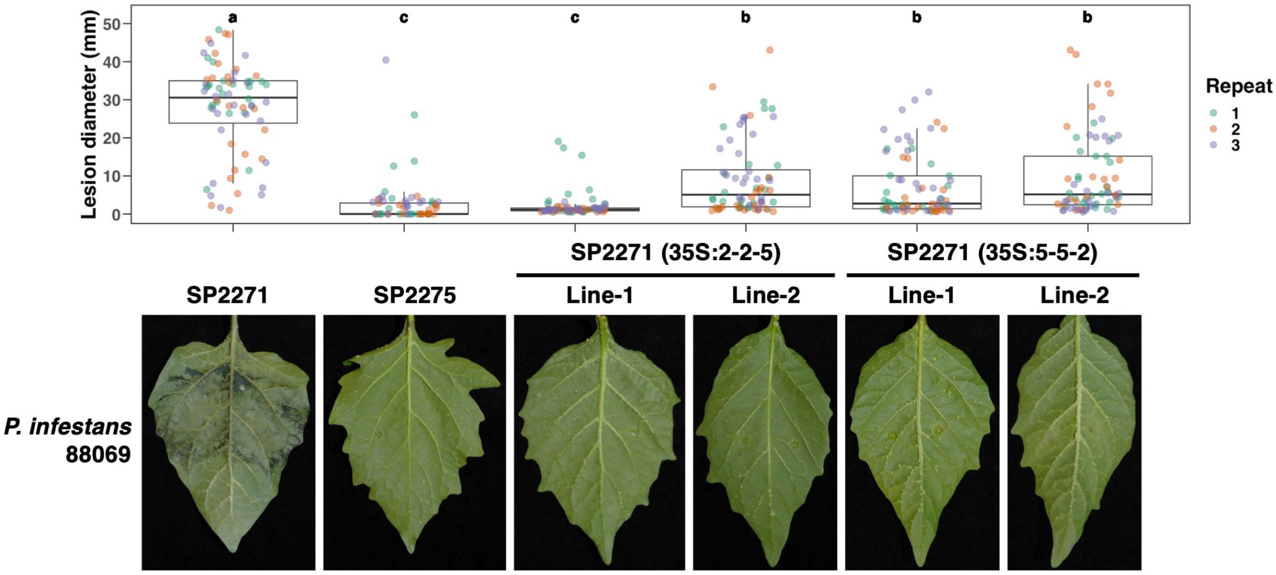

Supplementary figure 19. Stable transformation of *S. americanum* demonstrates that Rpi-amr5/Rpc2 domain swaps are functional and confer resistance to *P. infestans.* The 5-5-2 and 2-2-5 chimeras both confer resistance to *P. infestans* in stably transformed *S. americanum* (accession SP2271). Leaves were inoculated with 10 µl droplets of *P. infestans* strain 88069 (20,000 spores ml^{-1}). . At 7 days post-inoculation, lesion diameters were measured and leaves were imaged. Two independent transgenic events are shown for each construct. Three biological replicates were performed; all data points (72 per treatment) are presented as box-and-whisker plots. Statistical differences were determined using one-way ANOVA and Tukey's HSD test ($p < 0.05$).

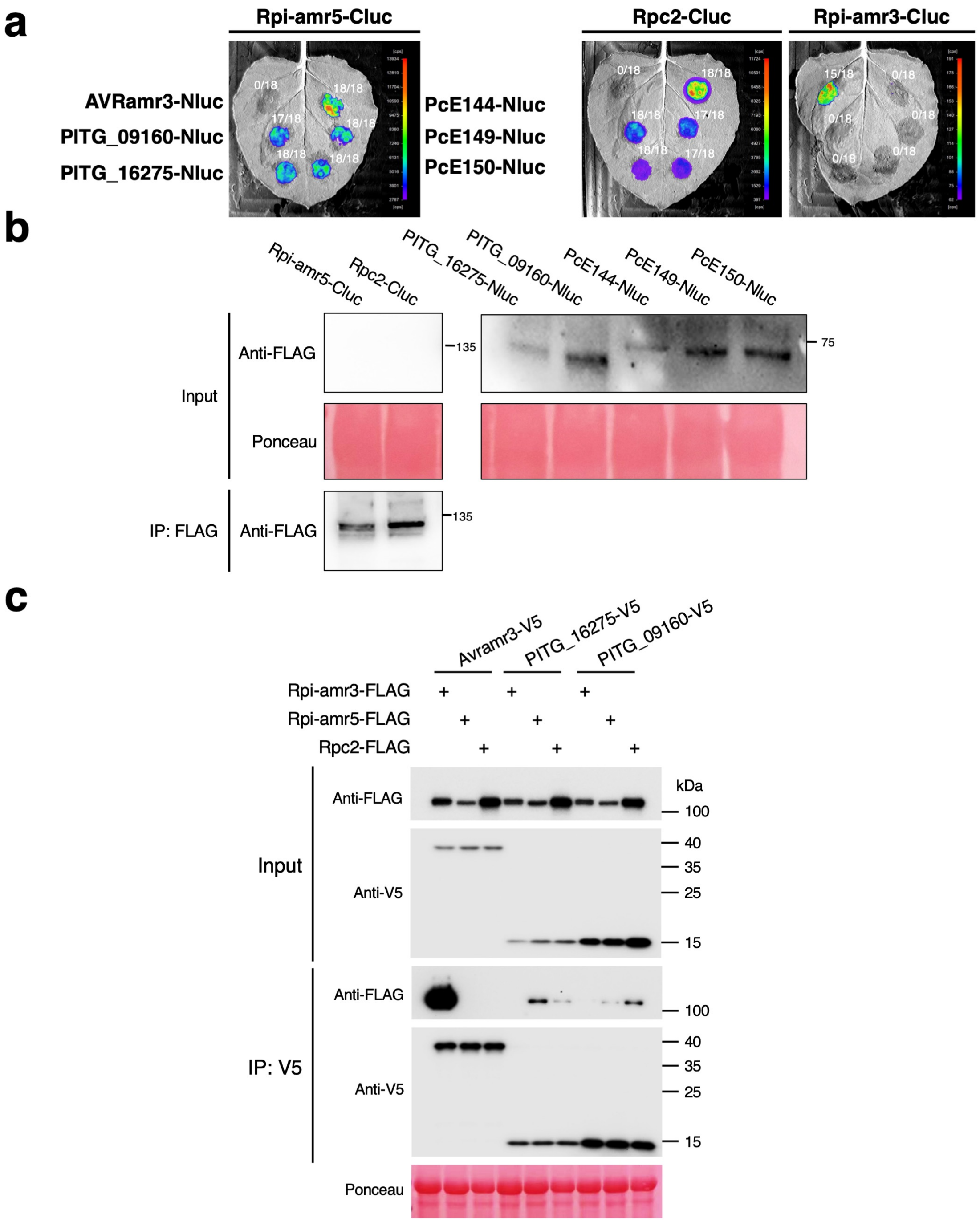

Supplementary figure 20. Split luciferase and co-immunoprecipitation assays demonstrate that Rpi-amr5 and Rpc2 associate with their cognate effectors *in planta.* (a) A split luciferase assay was used to test for *in planta* association between the five recognised effectors (PITG_09160, PITG_16275, PcE144, PcE149, and PcE150) and both Rpi-amr5 and Rpc2. Luminescence was observed when *Rpi-amr5*, or *Rpc2*, but not *Rpi-amr3*, was co-expressed with the effectors. To avoid cell death, the assay was performed in the *nrc2/3/4* KO *N. benthamiana* line. Sensor NLRs were tagged with a C-terminal luciferase fragment, and effectors with an N-terminal luciferase fragment. **(b)** Western blotting demonstrates the expression of constructs used in the split luciferase assay. *Agrobacterium* strains were infiltrated into whole *N. benthamiana* leaves at OD_{600} = 0.2, and leaves were harvested at 3 dpi. As expression of Rpi-amr5 and Rpc2 was low, samples were enriched by immunoprecipitation before reimaging. **(c)** *S. americanum* NLRs Rpi-amr5-FLAG and Rpc2-FLAG both co-immunoprecipitate with the *P. infestans* effectors PITG_09160-V5 and PITG_16275-V5. In contrast, Rpi-amr3-FLAG does not co-immunoprecipitate with either effector. AVRamr3 was used as a control, and Rpi-amr3-FLAG was successfully detected following an AVRamr3-V5 immunoprecipitation. Samples were prepared from agroinfiltrated leaves of the *nrc2/3/4 N. benthamiana* line. *Agrobacterium* strains were infiltrated at OD_{600} = 0.5, and samples were harvested at 2 dpi.

Supplementary table 1. Four NLR-encoding genes were identified as *Rpi-amr5* candidates. *Rpi-amr5* maps to the *Rpi-amr1* cluster on Chromosome 11. Of the 16 paralogs identified in the SP2275 reference genome, four are candidates for *Rpi-amr5*. The ID, whether expression was detected, and homology to the characterised *Rpi-amr1* cluster (from SP2273) is shown. Expression of each candidate was determined using cDNA RenSeq data from fully expanded leaves of SP2275 without pathogen infection. An absence of expression was defined by a complete absence of reads mapping to the candidate gene.

NLR_gene_ID	Expression in SP2275	Notes	Retained as a candidate?
NLR_1-2275	Expressed	**Premature stop preceding the LRR-domain**	**-**
NLR_2-2275	**No expression**	-	**-**
NLR_3-2275	Expressed	-	**Yes**
NLR_4-2275	**No expression**	-	-
NLR_5-2275	**No expression**	-	-
NLR_6-2275	Expressed	-	**Yes**
NLR_7-2275	Expressed	**Frameshift mutation leads to a premature stop within the NB-ARC domain**	-
NLR_8-2275	Expressed	**Predicted CDS does not contain a complete CC domain**	-
NLR_9 (Rpi-amr1h-2275)	Expressed	**Transient expression in *N. benthamiana* results in constitutive activity**	**Yes**
NLR_10-2275	Expressed	**Predicted CDS does not contain a complete CC domain**	-
NLR_11-2275	Expressed	**Frameshift mutation leads to a premature stop within the NB-ARC domain**	-
NLR_12-2275	Expressed	**Identical to non-*Rpi-amr5* accession, not tested**	-
NLR_13 (Rpi-amr1c-2275)	Expressed	**Transient expression in *N. benthamiana* results in constitutive activity**	**Functional Rpi-amr5**
NLR_14-2275	Expressed	**No Clear ORF and an incomplete NB-ARC domain**	-
NLR_15-2275	Expressed	**The predicted CDS encodes a CC and an incomplete NB-ARC domain, but no LRRs.**	-
NLR_16-2275	Expressed	**Identical to non-*Rpi-amr5* accession, not tested**	-

Supplementary table 2. Five NLR-encoding genes were identified as *Rpc2* candidates. Using bulked segregant analysis, *Rpc2* was mapped to the *Rpi-amr1*/*Rpi-amr5* cluster on Chromosome 11. Of the 8 paralogs identified within the *Rpc2* mapping interval, five were retained as candidates. The ID and homology to the characterised *Rpi-amr1* cluster is shown.

NLR_gene_ID	Candidate notes	Retained as a candidate?
NLR_1-2308 (Rpi-amr1h-2308)	-	**Yes**
NLR_2-2308 (Rpi-amr1l-2308)	-	**Yes**
NLR_3-2308 (Rpi-amr1e-2308)	-	**Yes**
NLR_4-2308	**Probably pseudogenised due to a 5 Kb insertion into NB-ARC**	-
NLR_5-2308 (Rpi-amr1c-2308)	**Transient expression in *N. benthamiana* results in constitutive activity**	**Functional Rpc2**
NLR_6-2308	**Predicted CDS does not encode a complete NB-ARC domain**	-
NLR_7-2308 (Rpi-amr1a-2308)	**Transient expression in *N. benthamiana* results in constitutive activity**	**Yes**
NLR_8-2308	**No clear ORF observed, indicating absence of start codon or presence of a premature stop**	-

Supplementary table 3. Effectors recognised by Rpi-amr1e-2308, Rpi-amr5, or Rpc2. *P. capsici* and *P. infestans* effectors were screened to identify elicitors of Rpi-amr1e-2308, Rpi-amr5 and Rpc2. Seven effectors were identified in this study, six recognised by either Rpi-amr5 or Rpc2, and a *P. capsici* homolog of AVRamr1 (PcapAVRamr1) that is recognised by both Rpi-amr1e-2273 and Rpi-amr1e-2308. These effectors were identified through a screen in S. americanum accessions SP2275 and SP2308, effectors that elicit an HR were co-expressed with Rpi-amr5 and Rpc2 in *N. benthamiana* to identify the true elicitors, and with Rpi-amr3 as a negative control. This assay, presented in Fig. 4 was repeated three times. The ID (PITG for *P. infestans* effectors, PcE for *P. capsici* effectors), the gene which encodes the effector, and the amino acid sequence of each effector (after the EER motif) is shown below.

Effector ID	Gene ID	Recognised by	Amino acid sequence post-EER
PITG_09160	PITG_09160	Rpi-amr5, Rpc2	VNAQKEAAAKVLDQVFKTKSLSPLDK LEKTSNLAVIRHVAAMVDDKVDKIFAF ADAVGMGRASMLKMLKGDKQFTDAE RLKTVKRYVKFLIKKEQNNKA
PITG_16275	PITG_16275	Rpi-amr5, Rpc2	GFKEVAEEAMAKFVVDKVYRTNSFSA LEKLEKTLNLPVLEKALVLADDKMKG AFKFAEEAKMNPADMAKIVKDFPDFT DAEKMKTVLEYTNYLKTMGKLD
PcE99	DVH05_000420	Rpc2	AGPQLVKSLSEKFSKKAAEKLTRSKSF NDFKKLDDVAYKENFHSATQALYQRIE KMGFNPDGMLKTMRKRGDVDLDLLA EYTRYWMRKYPTWTKNQ
PcE144	DVH05_026132	Rpi-amr5, Rpc2	IGTQFITSLSDKFASAADKLTRSKSLNSI SKVDDIAPGRAGKTLGEVLKIDDVAYL KAAGKWSKNLDDVEDAKLVSELSKLD HKEALRVLDEKNAAAFKSIEDMGYSPD GMFKKMKELGKDYFDPFLLKHYTRYW MAKHPTWTSSL
PcE149	DVH05_000363	Rpi-amr5, Rpc2	TGPQFIKSLSEKFAKAAETGNFAKTVEK LTRSKSLNGISKVDDVVVPGRAGKTLT EVLKIDDVAYLKAAGKWPKNLDDIQE AKLVREISTLDDQATLKLITKENAAEFK KIEDMGFNPDGMLKRMKQLSKNDKDY FDLVLLKHYTQYYMAKHPTWASSLG
PcE150	DVH05_026158	Rpi-amr5, Rpc2	TGPQFIKSLSEKFAKAAETGKFAKTVEK LTRSKSLNGISKVDDVVAPGRAGKTLS EVLKIDDVAYLKAAGKWPKNLDDIDE AKLVREISTLDDQATLKHITKENAAEF KKIEGMGYNPDGMLKKMKQLSKDVD KDYYDLILLKHYTQYYMAKHPTWASS L
PcE173 (PcapAVRamr1)	DVH05_027339	Rpi-amr1e-2308, Rpi-amr1e-2273	LAGKNMFNAEKIEKALQDTSYAKTLFR RWKRYEVEHGAAFDKLIKFNIGKDDK VFGLYKSYVSWLEKHHPLGAETGGGP NLFSKAKLDKAMKDPKYENTMFGRW KRQGFESDAAYNKLLAFNLASDADVY KIYTKYVTWLNIHHPLAKTRKTTAKDF LFNVDRIARAKKDSEFAETLFKKWKTS GLDEKPVYLKLWDMGLKTDDELYKLY KNYVKWLDIHYPLPAKAT

Supplementary table 4. NLR-encoding genes characterised in this study. Three novel NLR-encoding genes were characterised in this study - *Rpi-amr1e-2308*, *Rpi-amr5* and *Rpc2*. The gene names, reference sequence, and accession from which they were cloned is detailed below. Sequence references are as described by Lin et al 2023, and are available at https://figshare.com/projects/The_Solanum_americanum_pangenome_and_effectoromics_reveals_new_resistance_genes_against_potato_late_blight/145449

***R*-gene**	**Cloned from accession**	**Reference contig**	**Gene ID/position**
Rpi-amr5	SP2275	utg376	313,074-318,083
Rpi-amr1e-2308	SP2308	Contig_24_SP2308_SMRT_RenSeq_RH_5_20__1	nlr_3
Rpc2	SP2308	Contig_4_SP2308_SMRT_RenSeq_RH_5_20__1	nlr_2
